# β-Integrin de-phosphorylation by the Density-Enhanced Phosphatase DEP-1 attenuates EGFR signaling in *C*. *elegans*

**DOI:** 10.1371/journal.pgen.1006592

**Published:** 2017-01-30

**Authors:** Michael Walser, Christoph Alois Umbricht, Erika Fröhli, Paolo Nanni, Alex Hajnal

**Affiliations:** 1 Institute of Molecular Life Sciences, University of Zürich, Winterthurerstr. 190, University of Zürich, Zürich, Switzerland; 2 Molecular Life Science Zürich PhD program, Zürich, Switzerland; 3 Functional Genomics Center Zürich, University of Zürich/ETH Zürich, Winterthurerstr. 190, Zürich, Switzerland; University of California San Diego, UNITED STATES

## Abstract

Density-Enhanced Phosphatase-1 (DEP-1) de-phosphorylates various growth factor receptors and adhesion proteins to regulate cell proliferation, adhesion and migration. Moreover, *dep-1/scc1* mutations have been detected in various types of human cancers, indicating a broad tumor suppressor activity. During *C*. *elegans* development, DEP-1 mediates binary cell fate decisions by negatively regulating EGFR signaling. Using a substrate-trapping DEP-1 mutant in a proteomics approach, we have identified the *C*. *elegans* β-integrin subunit PAT-3 as a specific DEP-1 substrate. DEP-1 selectively de-phosphorylates tyrosine 792 in the membrane-proximal NPXY motif to promote integrin activation via talin recruitment. The non-phosphorylatable β-integrin mutant *pat-3(Y792F)* partially suppresses the hyperactive EGFR signaling phenotype caused by loss of *dep-1* function. Thus, DEP-1 attenuates EGFR signaling in part by de-phosphorylating Y792 in the β-integrin cytoplasmic tail, besides the direct de-phosphorylation of the EGFR. Furthermore, *in vivo* FRAP analysis indicates that the αβ-integrin/talin complex attenuates EGFR signaling by restricting receptor mobility on the basolateral plasma membrane. We propose that DEP-1 regulates EGFR signaling via two parallel mechanisms, by direct receptor de-phosphorylation and by restricting receptor mobility through αβ-integrin activation.

## Author summary

The phosphorylation of proteins by kinases is one of the most common post-translational modifications that regulates protein function in a variety of processes. Protein kinases are usually counteracted by specific phosphatases that remove the phosphate groups from proteins. We have undertaken a systematic biochemical (“proteomics”) approach to identify the substrates of the Density-Enhanced Phosphatase DEP-1. The DEP-1 phosphatase has previously been identified as a growth inhibitor and tumor suppressor that de-phosphorylates different growth factor receptors to inhibit intracellular signal transduction and cell proliferation. Here, we have identified a ß-integrin subunit as a specific DEP-1 substrate. Integrins consist of one α and one β subunits that function as cell adhesion molecules mediating the attachment of cells to the extracellular matrix. The de-phosphorylation of integrins causes their activation and stabilizes the adhesion sites. By engineering a ß-integrin mutation that cannot be phosphorylated and hence does not depend on the DEP-1 phosphatase, we show that integrin activation not only permits extracellular matrix adhesion but also attenuates epidermal growth factor signaling. Our findings point at a dual role of the DEP-1 phosphatase in regulating EGFR signaling by simultaneously de-phosphorylating integrins and growth factor receptors.

## Introduction

Protein phosphorylation is one of the most common post-translational modifications used by eukaryotic cells to regulate various aspects of protein function. Signaling through the conserved Epidermal Growth Factor Receptor (EGFR) pathway involves receptor auto-phosphorylation as well as the phosphorylation of several downstream signal transduction molecules once an EGF ligand has bound to and activated the receptor tyrosine kinase [1]. On the other hand, protein phosphatases are key components of inhibitory networks that attenuate EGFR signaling before and after ligand binding [2]. The genomes of vertebrates and invertebrates encode a large number of predicted phosphatase genes, of which many are implicated in human diseases [3]. However, the physiological substrates of most protein phosphatases are not well defined, and it is often difficult to correlate a specific phosphatase activity with *in vivo* changes in protein phosphorylation [2].

**Fig 1:**
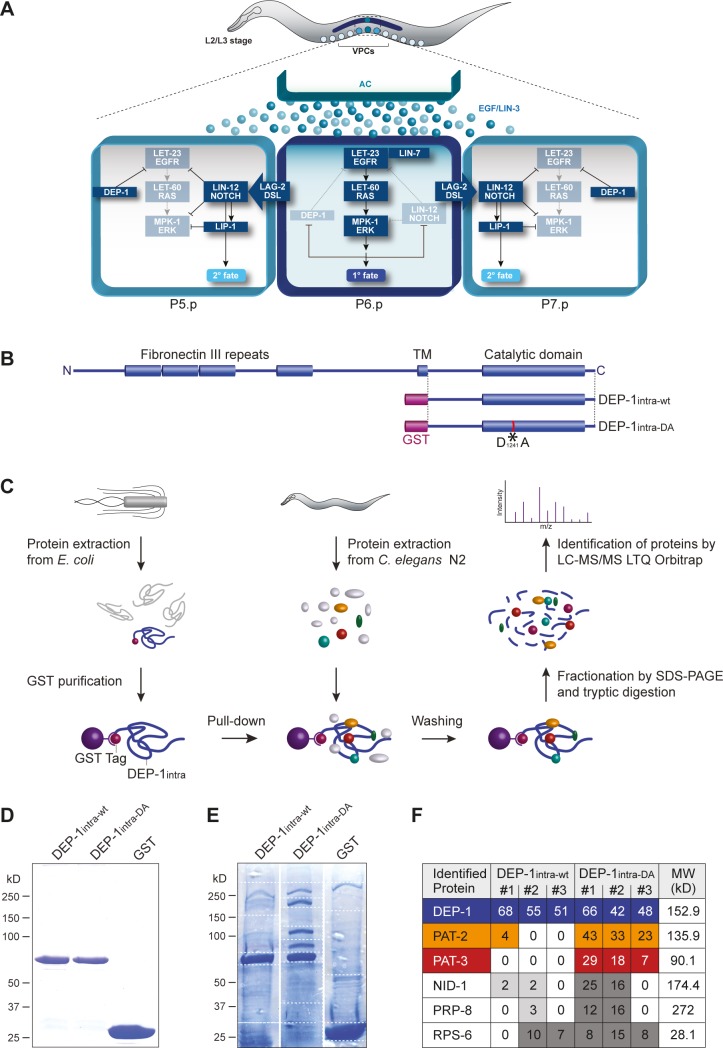
A mass spectrometry-based approach identifies the β-integrin PAT-3 as a DEP-1 substrate. (A) Model for DEP-1 function during vulval development. The AC induces the EGFR/RAS/MAPK pathway to specify the 1° cell fate in P6.p. The 1° P6.p cell then produces a lateral LAG-2/DSL signal that activates the LIN-12 NOTCH pathway in the adjacent VPCs P5.p and P7.p to specify the 2° cell fate. EGFR/RAS/MAPK signaling represses DEP-1 and NOTCH expression in the 1° VPC, while DEP-1 and LIP-1 block EGFR/RAS/MAPK signaling in the 2° VPCs by de-phosphorylating the EGFR and MAPK, respectively. (B) Domain structures of *C*. *elegans* DEP-1 and schematic of GST-tagged DEP-1intra-wt and DEP-1intra-DA fusion proteins. The asterisk indicates the position of the substrate trapping mutation D1241A. (C) Workflow of GST pull-down and subsequent analysis by LC-MS/MS to identify DEP-1 binding proteins. (D) Samples of purified DEP-1intra-wt, DEP-1intra-DA and GST proteins used for the pull-down were loaded on an SDS gel and stained with Coomassie Blue. (E) Eluted proteins after pull-down from N2 wild-type protein extracts with purified GST::DEP-1 intra-wt, GST::DEP-1intra-DA, or GST were fractionated by SDS-PAGE on separate gels and stained with Colloidal Coomassie Blue. Dashed lines indicate the cutting lines to fractionate the SDS gels for subsequent tryptic in-gel digestion. (F) Top-six proteins that were identified by LC-MS/MS in three independent pull-down experiments. The numbers of identified peptides per protein are shown for each experiment. All listed proteins were not detected in the GST only controls. For the complete list of identified peptides, see S1 Table. Protein identification threshold was set to 95.0% probability by the Scaffold Proteome Software and contained at least 2 exclusive uniquely identified peptides.

The Density Enhanced Phosphatase DEP-1, also known as PTPRJ, PTP-η or CD148, belongs to the class III Receptor Protein Tyrosine Phosphatase (R-PTP) family [4,5]. Like the other R-PTPs of this family, DEP-1 contains a single intracellular catalytic tyrosine phosphatase domain, a transmembrane domain, and multiple extracellular fibronectin type III repeats (Fig 1B). DEP-1 was originally isolated as a phosphatase whose expression is up-regulated in contact-inhibited, dense cell cultures [5]. The mouse *dep-1* gene was independently identified as the colon cancer susceptibility locus *Scc1*, and human *dep-1* is frequently deleted or mutated in various cancer types, such as thyroid, colon, lung, pancreatic, and breast cancer [2,6]. DEP-1 inhibits cell motility in contact-inhibited cell cultures and exhibits tumor-suppressor activity when overexpressed in cancer cells [7–10]. A multitude of potential DEP-1 substrates have been identified, including various growth factor receptors such as EGFR, PDGFR, VEGFR, FLT-3, MET, the ERK-2 kinase, the p85 subunit of PI3K as well as cell-cell junction proteins like p120^ctn^, β-catenin and γ-catenin, occludin and ZO-1 [4,11,12].

We have previously identified the *C*. *elegans dep-1* ortholog in a forward genetic screen for negative regulators of the EGFR/RAS/MAPK signaling pathway [11]. DEP-1 controls cell fate decisions by inhibiting the activation of the EGF receptor (termed LET-23 in *C*. *elegans*) in different tissues. Most prominently, *dep-1* is required for a binary cell fate switch during vulval development. Towards the end of the second larval stage, the uterine anchor cell (AC) secretes the EGF-like growth factor LIN-3 to induce the differentiation of the adjacent vulval precursor cells (VPCs) P3.p to P8.p [13]. All six VPCs express LET-23 EGFR on their basolateral membrane, but the VPC that is located closest to the AC (P6.p) sequesters most of the LIN-3 EGF signal and responds by adopting the primary (1°) vulval cell fate (Fig 1A). P6.p then produces a lateral DELTA signal that activates the NOTCH signaling pathway in the adjacent VPCs P5.p and P7.p. NOTCH signaling in these VPCs induces the expression of several inhibitors of the EGFR/RAS/MAPK pathway, such as the MAPK phosphatase LIP-1, to inhibit the 1° and induce the alternate secondary (2°) cell fate [14–16]. In this context, DEP-1 is part of a negative feedback loop, in which EGFR signaling down-regulates DEP-1 expression in the 1° VPC, while NOTCH signaling maintains high DEP-1 expression in the 2° VPCs (Fig 1A). As a consequence of this signaling cross-talk, the EGFR remains fully active in the 1° VPC, but is repressed by DEP-1 in the 2° VPCs. Genetic epistasis experiments indicated that DEP-1 inhibits signaling at the level of the EGFR and biochemical studies indicated that the EGFR is a DEP-1 substrate [11].

To explore new functions of DEP-1 in *C*. *elegans*, we searched for additional DEP-1 substrates through a proteomics approach and thereby identified the β-integrin subunit PAT-3. Integrins are type I transmembrane glycoproteins that function as cell adhesion molecules and form heterodimers consisting of one α and one β subunit. The *C*. *elegans* genome encodes a single β-subunit, PAT-3, and two α-subunits, PAT-2 and INA-2 (INtegrin Alpha). *pat-2* or *pat-3* loss-of-function mutations cause a “Paralyzed and Arrested at Two-fold” embryonic lethal (PAT) phenotype because PAT-2 and PAT-3 are essential to attach the body wall muscles to the overlaying epidermis during elongation of the embryo [17]. Mutations in *ina-1*, on the other hand, cause larval lethality with pleiotropic defects, as the INA-1/PAT-3 complex is required for the correct migration of various cell types [18].

Integrins play important roles in transducing extra- and intra-cellular signals in either direction, in what is referred to as either “inside-out” or “outside-in” signaling [19–21]. In their active, open conformation, integrins bind to a variety of cell-surface, extracellular matrix (ECM) or soluble protein ligands. The phosphorylation status of the β-integrin cytoplasmic tail controls the recruitment of different cytoplasmic adaptor proteins to focal adhesion sites. In particular, the cytoplasmic FERM domain protein talin, called TLN-1 in *C*. *elegans*, binds preferentially to the un-phosphorylated membrane-proximal NPXY_792_ motif in activated β-integrins, thereby connecting the F-actin cytoskeleton to the αβ-integrin complex [22]. The membrane-distal NPXY motif on the other hand, binds to kindlin and regulates intracellular integrin trafficking [19,21,22].

The *C*. *elegans* integrins have so far not been implicated in the regulation of the EGFR/RAS/MAPK signaling pathway. Though, there are several reports of cross-talk between integrins and the EGFR signaling pathway in mammalian cells [23].

## Results

### A mass spectrometry-based approach identifies the β-integrin PAT-3 as a DEP-1 substrate

In order to identify additional substrates of *C*. *elegans* DEP-1, we undertook a mass spectrometry-based approach, in which proteins enriched through pull-down experiments were analyzed by LC-MS/MS. For this purpose, the intracellular domain of DEP-1 carrying the substrate trapping mutation D1241A (DEP-1intra-DA, Fig 1B) [11] was expressed as a GST fusion protein in *E*. *coli*, affinity-purified on glutathion-sepharose and incubated with total protein extracts of mixed-stage *C*. *elegans* wild-type N2 animals (Fig 1C and 1D and materials and methods). To distinguish between DEP-1 substrates that specifically bound to the substrate-trapping mutant and other DEP-1 binding proteins, we performed parallel pull-down experiments with the wild-type intracellular domain (DEP-1intra-wt) and with GST alone as negative control (Fig 1B and 1D). Protein complexes were separated by SDS-PAGE and stained with colloidal Coomassie-blue. We identified three prominent protein bands besides the DEP-1intra-DA bait with molecular weights of around 90 kD, 130 kD, and 170 kD that were present exclusively in the DEP-1intra-DA pull-down (Fig 1E). After fractionation of the SDS-gel (dotted lines in Fig 1E), proteins were processed for in-gel tryptic digestion and C18 ZipTip purification. Liquid chromatography and tandem mass spectrometry (LTQ Orbitrap LC-MS/MS) in conjunction with a database search against a *C*. *elegans* proteome led to the identification of 585 proteins in three independent pull-down experiments. Ninety-seven of the identified proteins were not present in the GST negative control (Fig 1F, see S1 Table for the full list). Among the 50 proteins (represented by 404 peptides) that bound preferentially to DEP-1intra-DA, 99 peptides were assigned to the α-integrin subunit PAT-2 (MW = 135.9 kD) and 54 peptides to the β-integrin subunit PAT-3 (MW = 90.1 kD) (Fig 1F). Furthermore, 41 peptides were from NID-1 (MW = 174.4 kD), an ECM component that interacts with the extracellular domain of integrins [24]. Since PAT-2 and PAT-3 peptides were found in each of the three replicate experiments using DEP-1intra-DA, the α-integrin and the β-integrin are the strong candidates for DEP-1 substrates. Although we have previously reported that DEP-1intra-DA specifically interacts with the EGFR homolog LET-23 [11], LET-23 was not present among the 50 proteins that preferentially bound to DEP-1intra-DA, presumably because LET-23 protein abundance in total animal extracts is too low to be detected in this assay (bottom 5% of all proteins) [25].

Tyrosine phosphorylation of the intracellular domain of the β-integrin subunits is known to regulate integrin activity [20,21], but tyrosine phosphorylation of the α subunits has so far not been reported. We thus hypothesized that DEP-1 de-phosphorylates PAT-3 in a complex with PAT-2.

### DEP-1 de-phosphorylates the membrane-proximal NPXY_792_ motif in the PAT-3 β-integrin cytoplasmic tail

**Fig 2:**
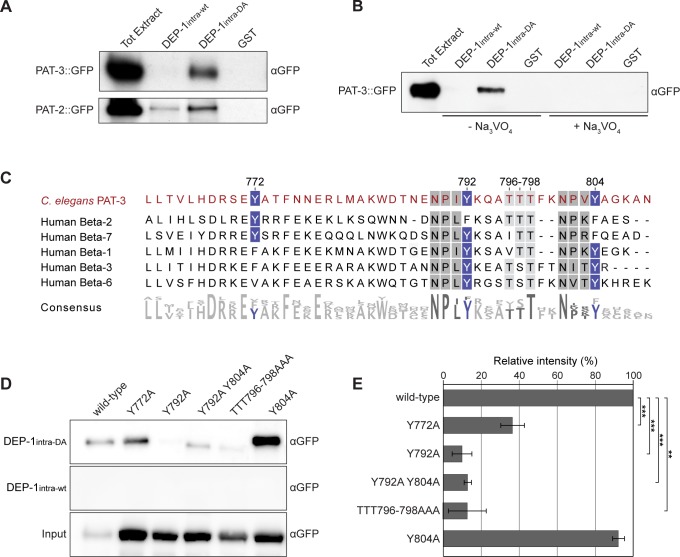
DEP-1 de-phosphorylates the NPXY_792_ motif in PAT-3. (A) Binding of PAT-3::GFP and PAT-2::GFP to DEP-1intra-DA. Total proteins extracts from the *pat-3*::*gfp* strain *qIs43* (top panel) and the *pat-2*::*gfp* strain *jeIs2222* (bottom panel) were incubated with the indicated GST fusion proteins and bound proteins were detected on anti-GFP Western blots. The Coomassie blue stained gels showing the purified GST fusion proteins used for this assay are shown in S1 Fig. (B) The interaction of DEP-1intra-DA with PAT-3::GFP is inhibited by the addition of 5 mM Na_3_VO_4_. (C) Sequence alignment of the *C*. *elegans* PAT-3 and human β2, β7, β1, β3, and β6 integrin cytoplasmic tails. Conserved tyrosine residues are highlighted in blue and NPXY domains are marked in grey. (D) Interaction of DEP-1intra-DA and DEP-1intra-wt with different PAT-3::GFP mutants containing Tyr to Ala or Thr to Ala exchanges. (E) Quantification of the binding experiments shown in (D). For each mutant construct, the average binding measured in three independent experiments is shown. Intensities of the bands were determined by densitometry, normalized to the input bands (bottom panel) and the values are shown as % of the binding detected with wild-type PAT-3::GFP. p-values are indicated with *** p<0.001 and ** p<0.01 in a two tailed student’s t-test—two-sample unequal variance. Error bars show the standard error of the mean.

To further characterize the binding of DEP-1intra-DA to PAT-3 and PAT-2, we performed pull-down experiments using purified DEP-1intra proteins and total protein extracts from worms expressing functional PAT-3::GFP or PAT-2::GFP transgenes [26,27]. Western blotting with anti-GFP antibodies revealed a specific interaction of DEP-1intra-DA with PAT-3::GFP, whereas no binding of DEP-1intra-wt to PAT-3 was detected (Fig 2A, top). Similar results were obtained with total protein extract from PAT-2::GFP expressing worms. PAT-2::GFP did interact with DEP-1intra-DA, though a weaker interaction was also observed with DEP-1intra-wt (Fig 2A, bottom). To test whether the interaction between PAT-3 and DEP-1 depends on the catalytic phosphatase domain, we added the PTP inhibitor sodium orthovanadate (Na_3_VO_4_) to the protein extracts before performing the binding experiment [28]. The interaction of DEP-1intra-DA with PAT-3::GFP was abolished in the presence of 5 mM Na_3_VO_4_ (Fig 2B). Since only the substrate-trapping DA mutant of DEP-1 interacted with PAT-3 and this interaction was blocked by orthovanadate treatment, we conclude that PAT-3 is most likely a direct substrate of DEP-1.

The phosphatase domain of DEP-1 is located in the cytoplasmic region, indicating that DEP-1 de-phosphorylates a phospho-tyrosine residue in the cytoplasmic tail of PAT-3. The PAT-3 intracellular domain contains three conserved tyrosine residues, Y772, Y792, and Y804 (Fig 2C). Y792 and Y804 are part of two conserved NPXY motifs, whose tyrosine phosphorylation regulates integrin activation and the recruitment of cytoplasmic adaptor proteins to the plasma membrane [17,21,22]. However, the protein purification and LC-MS/MS conditions we used did not allow the detection of phospho-peptides.

To determine, which of these tyrosine residues are recognized by DEP-1, we generated mutant *pat-3*::*gfp* transgenes, in which the cytoplasmic tyrosines Y772, Y792, Y804, as well as the conserved threonine triplet TTT796-798 located between Y792 and Y804 were replaced with alanines (Y772A, Y792A, Y792A Y804A, Y804A, and TTT796-798AAA). The interaction of DEP-1intra-DA with PAT-3 containing either the Y792A mutation in the membrane-proximal NPXY motif alone or in combination with the Y804A mutation in the membrane-distal NPXY motif was significantly reduced (Fig 2D and 2E). By contrast, the Y804A single mutation did not affect the binding of DEP-1intra-DA to PAT-3. Furthermore, the TTT796-798AAA mutation reduced the interaction of PAT-3 with DEP-1intra-DA, possibly because this triple mutation in the vicinity of the NPXY_792_ motif prevents substrate recognition by DEP-1.

Taken together, the protein interaction experiments suggest that DEP-1 selectively de-phosphorylates tyrosine Y792 in the membrane-proximal NPXY motif in the cytoplasmic tail of PAT-3.

### Mutation of the PAT-3 NPXY_792_ phosphorylation site attenuates EGFR/RAS/MAPK signaling

We have previously identified *dep-1* as a negative regulator of vulval induction [11]. Epistasis analysis indicated that DEP-1 negatively regulates EGFR/RAS/MAPK signaling by inhibiting LET-23 EGFR in the vulval precursor cells (VPCs). We thus investigated whether the de-phosphorylation of PAT-3 β-integrin by DEP-1 mediates the inhibitory activity DEP-1 exerts on EGFR signaling. For this purpose, we used CRISPR/Cas9-mediated genome editing to create a non-phosphorylatable *pat-3(Y792F)* allele, in which the Y792 phosphorylation site was replaced with Phenylalanine (F) (*pat-3(zh105)*, see materials and methods). To exclude potential off-target effects, we sequenced all predicted off-target sites with a score of at least 0.2 [29], but found no additional mutations at these sites (S2 Table). Furthermore, the *pat-3(zh105)* allele was backcrossed four times to the wild-type N2 strain to remove unlinked mutations before analysis.

**Fig 3:**
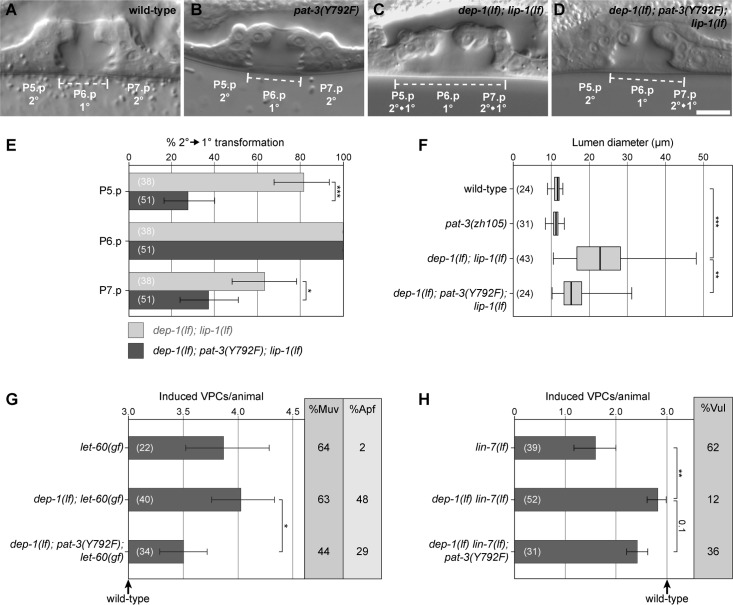
The *pat-3* Y792F mutation suppresses the *dep-1(lf)* phenotypes. (A) Nomarski images of wild-type, (B) *pat-3(Y792F)*, (C) *dep-1(lf); lip-1(lf)* and (D) *dep-1(lf); pat-3(Y792F); lip-1(lf)* mutant L4 larvae. The dashed lines indicate the diameter of the vulval lumen. Note in (C) the detachment of the P5.p and P7.p descendants, which is characteristic of a 2° to 1° fate transformation. The scale bar in (D) represents 10 μm. (E) Frequency of the 2° to 1° fate transformations in *dep-1(lf); lip-1(lf)* double versus *dep-1(lf); pat-3(Y792F); lip-1(lf)* triple mutants as exemplified in (C). (F) Box plot showing the lumen diameters in the indicated genotypes. (G) Vulval induction (VI) in *let-60(gf)* mutants combined with *dep-1(lf)* and *pat-3(Y792F)*. The bar chart shows the VI for each genotype, %Muv indicates the fraction of animals with a VI>3 and %Apf the fraction of animals with 2° to 1° fate transformations in the P5.p or P7.p descendants. (H) Vulval induction in *lin-7(lf)* mutants combined with *dep-1(lf)* and *pat-3(Y792F)*. The bar chart shows the VI for each genotype and %Vul indicates the fraction of animals with a VI<3. The error bars indicate the 95% confidence intervals, and the values in brackets refer to the numbers of animals scored. p-values are indicated with *** p<0.001, ** p<0.01 and * p<0.05. In (E), (G) and (H), the 95% confidence intervals and p-values were derived by bootstrapping 1000 samples, and p-values were Bonferroni corrected in (G) and (H). In (F), the p-values indicate the significances determined with ANOVA followed by Bonferroni correction.

**Fig 4:**
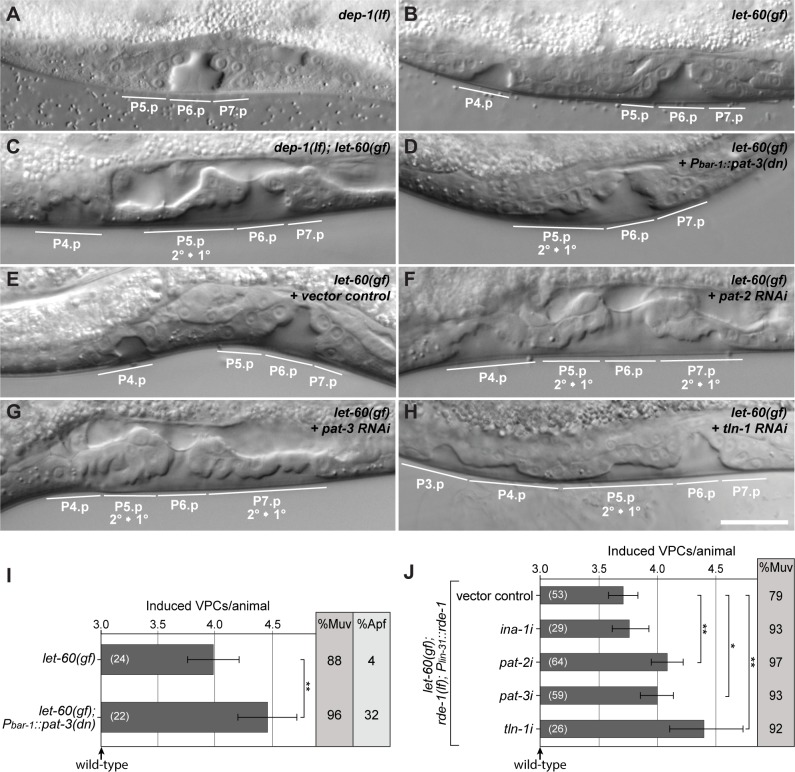
PAT-2, PAT-3 and TLN-1 negatively regulate EGFR/RAS/MAPK signaling. (A) Nomarski images of *dep-1(lf)*, (B) *let-60(gf)* single and (C) *dep-1(lf); let-60(gf)* double mutant larvae at the L4 stage. Note the detachment of the P5.p descendants indicating a 2° to 1° fate transformation and the ectopic invagination formed by P4.p and P8.p descendants in (C). (D) Vulva-specific expression of dominant negative *pat-3* in *let-60(gf)* mutant larva at the L4 stage. (E-H) Pn.p cell-specific RNAi in the *let-60(gf); rde-1(lf); zhEx418[lin-31*::*rde-1]* background [34] using (E) vector control, (F) *pat-2*, (G) *pat-3*, and (H) *tln-1* dsRNA expressing bacteria. Animals are at the L4 stage. Note in (F-H) the broad vulval invaginations due to the detachment of the P5.p and P7.p descendants. The scale bar in (H) represents approximately 10 μm. (I) Pn.p cell-specific expression of a dominant negative PAT-3 transgene increases the VI of *let-60(gf)* animals and results in an Apf phenotype. (J) Quantification of vulval induction in the *let-60(gf); rde-1(lf); zhEx418[lin-31*::*rde-1]* background after RNAi against *ina-2*, *pat-2*, *pat-3* and *tln-1*. The bar charts show the VI for each genotype, %Muv indicates the fraction of animals with a VI>3. The error bars show the 95% confidence intervals derived by bootstrapping 1000 samples, and the values in brackets refer to the numbers of animals scored. p-values derived by bootstrapping are indicated with ** p<0.01 and * p<0.05, and they were Bonferroni corrected in (J).

*pat-3(Y792F)* single mutants are viable and exhibit a superficially wild-type morphology with normal levels of vulval induction (Fig 3A and 3B). *dep-1(lf)* single mutants also develop a wild-type vulva due to the redundancy between the various negative regulators of the EGFR/RAS/MAPK pathway (Fig 4A) [11]. However, combined with loss-of-function (*lf*) mutations in other negative regulators of EGFR/RAS/MAPK signaling, such as a *lf* mutation in the MAP kinase phosphatase *lip-1*, *dep-1(lf)* causes a frequent transformation of the 2° cell fate of the P5.p and P7.p descendants into a 1°-like fate, a so called Adjacent Primary Fate (Apf) phenotype (Fig 3C and 3E) [11]. The *dep-1(lf); lip-1(lf)* Apf phenotype is characterized by the detachment of the P5.p and P7.p descendants from the cuticle and an enlarged diameter of the vulval lumen (Fig 3C and 3F). The *pat-3(Y792F)* allele partially suppressed the Apf phenotype of *dep-1(lf); lip-1(lf)* double mutants (Fig 3D). In particular, the frequency of animals, in which the P5.p or P7.p descendants were transformed into a 1°-like fate, was significantly decreased by the *pat-3(Y792F)* allele (Fig 3E). The suppression of the Apf phenotype was further reflected by a reduced vulval lumen diameter in *dep-1(lf); pat-3(Y792F); lip-1(lf)* triple mutants compared to the *dep-1(lf); lip-1(lf)* double mutants (Fig 3F).

We next investigated the genetic interaction between *pat-3(Y792F)* and mutations that either increase or decrease the activity of the EGFR/RAS/MAPK pathway. First, we analyzed a gain-of-function mutation in the *let-60* gene, which encodes the sole *C*. *elegans* RAS family member [30]. *let-60 ras(gf)* single mutants contain on average 3.8 induced VPCs per animal (i.e. the Vulval induction Index VI = 3.8) and exhibit a penetrant Multivulva (Muv) phenotype due to the hyper-activation of the downstream MAPK pathway (Fig 3G). However, the RAS/MAPK pathway in the *let-60 ras(gf)* background remains sensitive to the upstream signal from the EGFR and to lateral inhibition by NOTCH [31]. Hence, *let-60(gf)* animals only rarely exhibit an Apf phenotype (Fig 3G). By contrast, *dep-1(lf); let-60(gf)* double mutants exhibit a slightly enhanced Muv (VI = 4.1) and a penetrant Apf phenotype (Fig 3G) [11]. The VI as well as the frequency of the Apf and Muv phenotypes were decreased in *dep-1(lf); pat-3(Y792F); let-60(gf)* triple mutants, pointing at an inhibitory effect of the *pat-3(Y792F)* allele on EGFR/RAS/MAPK signaling.

The *lin-7* gene encodes a PDZ domain adaptor protein that is required for basolateral localization of LET-23 and efficient receptor activation by the inductive LIN-3 EGF signal [32]. *lin-7(lf)* mutants exhibit a partial vulvaless (Vul) phenotype that is efficiently suppressed by *dep-1(lf)* (Fig 3H). Consistent with an inhibitory effect of PAT-3 on the EGFR signaling pathway, the introduction of the *pat-3(Y792F)* mutation into the *dep-1(lf) lin-7(lf)* background caused a slightly decreased VI (statistically not significant) and an increased penetrance of the Vul phenotype (Fig 3H).

Thus, the *pat-3(Y792F)* allele partially suppresses the effects of *dep-1(lf)* on mutations that either increase or decrease the activity of the EGFR signaling pathway. These results indicate that DEP-1 attenuates EGFR signaling in part by de-phosphorylating the Y792 residue in the PAT-3 β-integrin subunit. Since the *pat-3(Y792F)* mutation only partially reverted the *dep-1(lf)* phenotype, DEP-1 must inhibit EGFR signaling through additional, PAT-3 independent mechanisms. One alternate mechanism of DEP-1 action is probably be the direct de-phosphorylation of the EGFR [11,12].

### Reduced αβ-integrin activity causes elevated EGFR/RAS/MAPK signaling

We next examined the effects of reduced αβ-integrin activity on EGFR/RAS/MAPK signaling. Since constitutive *pat-2(lf)* or *pat-3(lf)* mutations cause embryonic or early larval lethality, we first expressed a dominant-negative *pat-3* mutant in the VPCs under the control of the *bar-1* promoter (*P*_*bar-1*_::*pat-3(dn)*) [27,33]. In the sensitized *let-60 ras(gf)* background, the *P*_*bar-1*_::*pat-3(dn)* transgene caused an increase in vulval induction and a similar Apf phenotype as observed in *dep-1(lf); let-60(gf)* mutants (Fig 4A–4D and 4I). In addition, we used the tissue-specific RNAi strain *let-60(n1046*^*gf*^*); rde-1(ne219*^*lf*^*); zhEx418[lin-31*::*rde-1]* to reduce *pat-2* and *pat-3* activity specifically in the Pn.p cells, which include the VPCs [34]. Pn.p cell-specific RNAi against both, *pat-2* and *pat-3*, increased the vulval induction in the *let-60(gf)* background when compared to vector control animals (Fig 4E–4G and 4J). (Note that the overall reduced VI in the Pn.p cell-specific RNAi strain is due to the *rde-1(lf)* background [34]). Moreover, the *pat-2* and *pat-3* RNAi treated L4 larvae showed a similar Apf phenotype as observed in *dep-1(lf); let-60(gf)* double mutants (Fig 4C). By contrast, RNAi of the second α-integrin subunit *ina-1* did not significantly change vulval induction in the *let-60(gf)* background (Fig 4J).

The conserved NPXY motifs in the β-integrin C-terminal tail serve as docking sites for adaptor proteins that mediate various cellular effects of the integrins. During integrin activation, the NPXY motifs in the β-integrin cytoplasmic tail are de-phosphorylated, thereby permitting the recruitment of the talin and kindlin adaptor proteins from the cytoplasm to the plasma membrane [22]. Talin binds specifically to the membrane-proximal NPXY_792_ motif of β-integrins and links the F-actin cytoskeleton to focal adhesion sites, while kindlin binds to the membrane distal NPXY motif that controls integrin trafficking. We therefore examined whether the single *C*. *elegans* talin ortholog TLN-1 mediates the inhibitory effect of PAT-2 and PAT-3 on the EGFR signaling pathway. *tln-1* RNAi in the *let-60(gf)* background caused a strong increase in the VI and similar vulval morphology defects as observed after Pn.p cell-specific *pat-3* or *pat-2* RNAi (Fig 4H and 4J). Thus, the PAT-2/PAT-3 αβ-integrin complex and its cytoplasmic adapter protein TLN-1 function as negative regulators of EGFR signaling during vulval development.

### DEP-1 regulates the recruitment of TLN-1 to the plasma membrane

**Fig 5:**
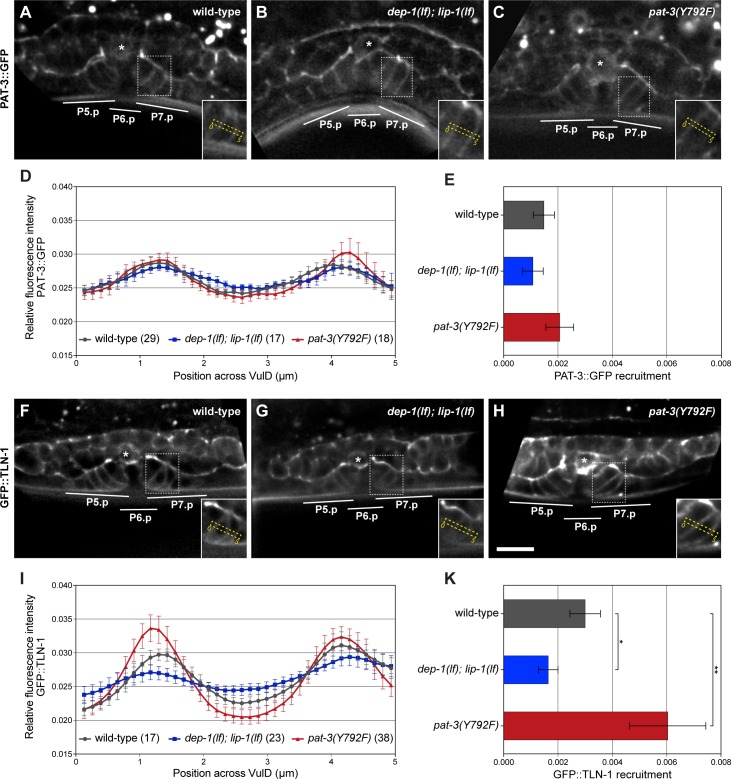
DEP-1 promotes the recruitment of TLN-1 to the basolateral plasma membrane. (A) Expression of the endogenous wild-type PAT-3::GFP reporter *zh115* in a wild-type and (B) a *dep-1(lf); lip-1(lf)* late L3 larva at the onset of vulval invagination. (C) Expression of the endogenous Y792F mutant PAT-3::GFP reporter *zh116* in a wild-type L3 larva. The insets in (A), (B) and (C) show the 2° VulD cells with the dashed yellow boxes indicating 5 μm long and 1.5 μm wide regions used to quantify PAT-3::GFP distribution. The asterisks indicate the position of the AC. (D) Average intensity profiles of the PAT-3::GFP signal in the regions shown in the insets of (A), (B) and (C). For each animal, the intensities were normalized to the summed intensities and the individual profiles were combined into the average profiles shown in (D), as described [35]. (E) Quantification of the membrane recruitment of PAT-3::GFP. The standard deviations of the mean intensities in the graphs shown in (D) were taken as a measure for uneven PAT-3::GFP distribution, indicating membrane recruitment [35]. (F) Expression of the endogenous GFP::TLN-1 reporter *zh117* in a wild-type, (G) a *dep-1(lf); lip-1(lf)* and (H) a *pat-3(Y792F)* mutant L3 larva. (I) Average intensity profiles of the GFP::TLN-1 signal in the regions shown in the insets of (F), (G) and (H), and (K) quantification of the membrane recruitment of GFP::TLN-1 were done as described above under (D) and (E). The error bars indicate the 95% confidence intervals, and the values in brackets the numbers of animals analyzed for each genotype. p-values in (K) determined with ANOVA followed by Bonferroni correction are indicated with ** p<0.01 and * p<0.05. The scale bar in (H) represents 10 μm.

According to our biochemical and genetic interaction studies, loss of *dep-1* function is predicted to cause the constitutive phosphorylation of the membrane-proximal NPXY_792_ motif in the PAT-3 β-integrin cytoplasmic tail. This may alter the intracellular trafficking of the αβ-integrin complex or its interaction with the cytoplasmic adaptor protein TLN-1 talin [20,22]. We therefore examined the subcellular localization of the CRISPR/Cas9 engineered endogenous PAT-3::GFP (*zh115*) and GFP::TLN-1 (*zh117*) reporters in the vulval cells of wild-type and *dep-1(lf); lip-1(lf)* larvae (see materials and methods). In addition, we engineered an endogenous Y792F mutant PAT-3(Y792F)::GFP reporter (*zh116*) to test if mutation of the phosphorylation site in the membrane-proximal NPXY_792_ motif altered PAT-3 localization. Wild-type PAT-3::GFP was expressed on the lateral membranes of the vulval cells and along the basal laminae that separate the vulval cells from the uterus (Fig 5A). Since PAT-3::GFP was also expressed in the adjacent uterine cells, both tissues are likely to contribute to the strong signal along the basal laminae. PAT-3::GFP localization on the lateral membranes of the vulval cells was most prominent at the onset of vulval invagination in mid to late L3 larvae (Fig 5A), allowing us to specifically observe PAT-3::GFP membrane recruitment in the vulval cells without the contribution from the ventral uterine cells that do not express DEP-1 [11]. To quantify the membrane localization of PAT-3::GFP, we generated intensity profiles from mid-sagittal optical sections across the 2° cells (Fig 5D) and calculated from these plots the standard deviation of the mean as a measure of membrane recruitment, as described previously [35] (Fig 5E). We then examined PAT-3::GFP localization in *dep-1(lf); lip-1(lf)* double mutants because the abnormal invagination of the 2° cells observed in this background (see Fig 3C) might be a result of reduced integrin activation. However, we did not observe an obvious change in PAT-3::GFP localization in the 2° vulval cells of *dep-1(lf); lip-1(lf)* mutants compared to wild-type larvae at the same stage (Fig 5B, 5D and 5E). Similarly, the localization of the mutant PAT-3(Y792F)::GFP reporter was not significantly changed compared to the wild-type PAT-3::GFP reporter (Fig 5C–5E). Thus, the de-phosphorylation of the membrane-proximal NPXY_792_ motif by DEP-1 did not appear to alter PAT-3 β-integrin localization.

We next examined the membrane recruitment of the TLN-1 talin adaptor protein in the different genetic backgrounds. The GFP::TLN-1 reporter showed a very similar expression pattern and subcellular localization as the PAT-3::GFP reporter, with prominent expression in all differentiating vulval and uterine cells. In addition to the basal and lateral plasma membrane-associated signal, GFP::TLN-1 expression was also observed in the cytoplasm of the vulval cells of wild-type L3 larvae (Fig 5F). In the *dep-1(lf); lip-1(lf)* background, GFP::TLN-1 exhibited a marked reduction of the signal on the lateral membranes and a more diffuse intracellular signal when compared to wild-type larvae at the same stage (Fig 5G and 5I). Quantification of the signal indicated a more uniform distribution of GFP::TLN-1 in *dep-1(lf); lip-1(lf)* mutants (Fig 5K). In addition, we examined GFP::TLN-1 localization in the *pat-3(Y792F)* mutant background. The recruitment of GFP::TLN-1 to the lateral VulD membrane was enhanced in the *pat-3(Y792F)* background compared to the wild-type background (Fig 5H–5K), suggesting that TLN-1 preferentially binds to unphosphorylated PAT-3, as reported for mammalian talin [22].

We conclude that DEP-1 promotes the membrane recruitment of TLN-1 in the 2° vulval cells by de-phosphorylating the NPXY_792_ motif in the PAT-3 β-integrin cytoplasmic tail, but DEP-1 has no measurable effect on PAT-3 localization.

### PAT-2/PAT-3 αβ-integrin and TLN-1 talin restrict EGFR mobility on the basolateral plasma membrane

**Fig 6:**
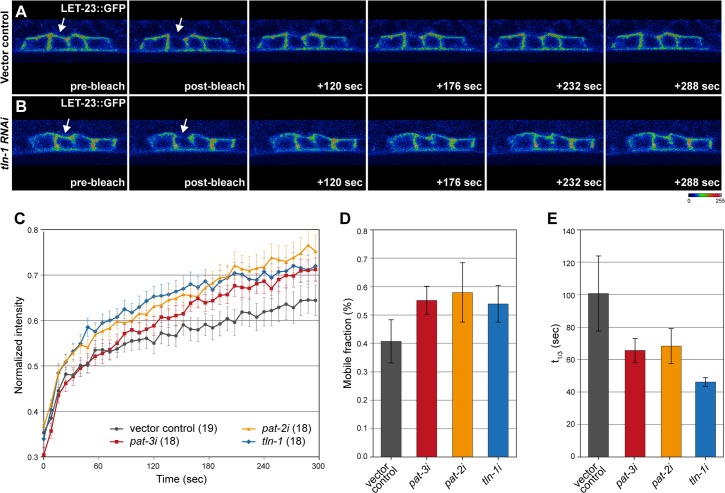
PAT-2/PAT-3 integrins and TLN-1 restrict EGFR mobility on the basolateral plasma membrane. (A) Examples from the FRAP analysis of LET-23::GFP in a vector control and (B) *tln-1* RNAi treated L3 larva at the Pn.pxx stage. The arrows in the pre- and post-bleach panels point at the basal membrane regions that were bleached. (C) Quantification of LET-23::GFP recovery after different RNAi treatments. The values on the y-axis show the LET-23::GFP signal intensity normalized to the intensity measured before bleaching in the bleached area and to the total signal intensity in the cell at each time point, and the values on the x-axis show the time after bleaching in seconds. The error bars indicate the standard error of the mean and the values in brackets the numbers of animals analyzed for each RNAi treatment. (D) Quantification of the relative (%) mobile fraction and (E) the t_1/3_ recovery time from the FRAP curves shown in (C). See S3 Table for the curve fitting analysis used to calculate these parameters. The error bars in (D) and (E) indicate the 95% confidence intervals.

To examine how the PAT-2/PAT-3 αβ-integrins and TLN-1 talin affect EGFR signaling, we measured LET-23 EGFR dynamics in the vulval cells after RNAi knock-down of the different components of the integrin complex. For this purpose, we performed *in vivo* fluorescence recovery after photobleaching (FRAP) experiments using a functional LET-23::GFP reporter, as previously described [34,36]. A portion of the basal membrane in the vulval cells at the Pn.px stage was bleached in *pat-2*, *pat-3* or *tln-1* RNAi treated animals, and the recovery of the LET-23::GFP signal was measured over a five minute period (Fig 6A and 6B). When compared to empty vector treated control animals, RNAi of *pat-2*, *pat-3* or *tln-1* resulted in an overall accelerated recovery of the LET-23::GFP signal (Fig 6C). We used a curve fitting algorithm to calculate the mobile receptor fraction (i.e. the fraction of the bleached LET-23::GFP signal that recovered after an infinite time period) and the recovery time t_1/3_, the time after which one third of the bleached signal had recovered, as described [34] (S3 Table). This analysis indicated that the faster recovery of LET-23::GFP after *pat-2*, *pat-3* or *tln-1* RNAi was due to an increase in the total mobile receptor fraction as well as to an increased receptor mobility reflected by the shorter t_1/3_ (Fig 6D and 6E).

The changes in LET-23 EGFR dynamics indicate that the PAT-2/PAT-3 αβ-integrins restrict the mobility of the receptor on the basal plasma membrane of the vulval cells together with TLN-1 talin. The increased mobility of LET-23 EGFR is consistent with the enhanced activation of the RAS/MAPK pathway after knock-down of integrin components (shown in Fig 4), as receptor trafficking is intimately coupled to downstream signaling [34].

## Discussion

### PAT-3 β-integrin is a physiological DEP-1 substrate

**Fig 7:**
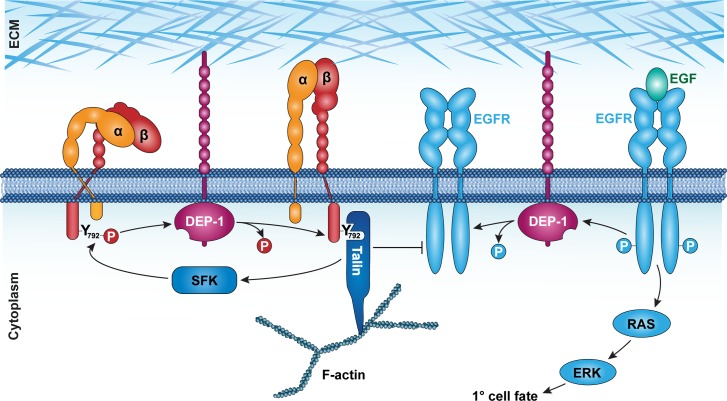
DEP-1 coordinates β-integrin activation with EGFR signaling. Left side: DEP-1 controls integrin activation by de-phosphorylating the membrane proximal NPXY_792_ residue in the β-integrin PAT-3. This promotes the recruitment of TLN-1 talin to focal adhesion sites, which attenuates EGFR trafficking and activity. Right side: DEP-1 directly de-phosphorylates the EGFR to inhibit RAS/MAPK activation.

DEP-1/PTPRJ was originally identified as a phosphatase that is up-regulated in confluent, contact-inhibited mammalian cell cultures [5]. Reduced DEP-1 activity results in enhanced phosphorylation and endocytosis of the EGFR, leading to increased cell proliferation [12]. Conversely, overexpression of DEP-1 causes rearrangements of the actin cytoskeleton, reduced focal adhesion kinase activity and defects in directed cell migration [7,37]. Although numerous DEP-1 substrates including the EGFR [11,12] and several other receptor tyrosine kinases have been identified, the relevant substrates that mediate the various effects DEP-1 exerts on cell proliferation and motility remain to be elucidated. By using the substrate-trapping DEP-1(D1241A) mutant in a proteomic approach, we have identified the β-integrin subunit PAT-3 as a novel DEP-1 substrate in *C*. *elegans*. DEP-1 shows a remarkable specificity for the membrane-proximal NPXY_792_ motif in the PAT-3 cytoplasmic tail, whereas the membrane-distal NPXY_804_ does not appear to be recognized by DEP-1. In order to test the physiological significance of this interaction, we engineered a non-phosphorylatable β-integrin mutant by replacing the codon encoding the Y792 residue in the membrane proximal NPXY motif with an F codon in the endogenous *pat-3 β-integrin* locus. Since the Y792F amino acid substitution in PAT-3 does not cause the embryonic lethal Pat phenotype observed in *pat-3(lf)* mutants [17], the Y792F mutant β-integrin likely forms active focal adhesion complexes together with PAT-2 α-integrin and retains its ability to interact with cytoplasmic adaptor proteins. However, the *pat-3(Y792F)* allele caused the partial reversion of the hyperactive EGFR signaling phenotype observed in *dep-1(lf)* mutants. This genetic interaction between *pat-3(Y792F)* and *dep-1(lf)* provides good evidence indicating that PAT-3 is indeed a physiologically relevant substrate of DEP-1. Since the *pat-3(Y792F)* mutation did not completely rescue the vulval phenotypes of *dep-1(lf)* mutants, the un-phosphorylated form of PAT-3 β-integrin mediates some but not all of the inhibitory effects of DEP-1 on EGFR signaling. Most likely, the direct de-phosphorylation of the EGFR accounts for the integrin-independent activity of DEP-1 [11]. We thus propose that DEP-1 acts via two redundant mechanisms to inhibit EGFR signaling (Fig 7).

### DEP-1 promotes integrin activation in the vulval cells

Phosphorylation of the NPXY motifs in the β-integrin cytoplasmic tail by Src family kinases (SFK) is a critical signal inducing a switch of the integrins from the active, open to the inactive, closed conformation, which results in a decreased adhesion to the ECM and increased cell motility [21]. Conversely, de-phosphorylation of the NPXY motifs promotes integrin activation and strengthens cell adhesion. To our knowledge, DEP-1 is the first protein phosphatase known to promote integrin activation via specific de-phosphorylation of a conserved NPXY motif.

Cytoplasmic talin proteins bind with their FERM domains preferentially to the un-phosphorylated, membrane-proximal NPXY_792_ motif in active integrin complexes and stabilize the open integrin conformation [21]. Talin binding strengthens the links between integrins and the cortical F-actin cytoskeleton, thereby stabilizing the focal adhesion complexes. The *pat-3(Y792F)* mutant can therefore be viewed as a constitutively open, active form of β-integrin that interacts with talin irrespective of SFK activity. The membrane-distal NPXY_804_ motif, on the other hand, binds to the integrin co-activator kindlin and regulates integrin recycling via sorting nexins [22]. Since DEP-1 specifically de-phosphorylates the membrane-proximal NPXY_792_ motif, DEP-1 promotes the recruitment of talin to the cell cortex. By contrast, DEP-1 does not appear to affect integrin localization, which involves the membrane-distal NPXY_804_ motif. Moreover, DEP-1 shows a highly tissue-specific expression pattern during *C*. *elegans* development, with most prominent expression in the vulval cells during the larval stages, in the excretory duct cell precursors in the embryo, and in a pair of sensory neurons in the head [11]. PAT-3 and PAT-2, on the other hand, are broadly expressed in many tissues during all stages of *C*. *elegans* development [17]. It thus seems likely that multiple protein phosphatases are required for integrin activation in different tissues. This probably explains why *dep-1(lf)* mutations neither cause a general loss of integrin activity nor a lethal PAT phenotype.

### The αβ-integrin-talin complex inhibits EGFR signaling

Components of the ECM were previously shown to bind to and stimulate the phosphatase activity of DEP-1 [38]. Thus, DEP-1 may be part of a focal adhesion complex that coordinates cell adhesion with receptor tyrosine kinase (RTK) signaling. Multiple modes of crosstalk between RTK pathways and focal adhesion complexes have been reported (reviewed by [23]). First, signal transduction by active integrins depends on intracellular signaling molecules that are shared with a number of receptor tyrosine kinase (RTK) signaling pathways, such as Src family kinases, SHC adapter proteins, focal adhesion kinase (FAK), Jun-dependent kinase, PKC and PI3K. For example, FAK and SHC are both downstream effectors of different RTK signaling pathways [39]. However, mutations in *kin-32*, which encodes the *C*. *elegans* FAK ortholog [40], or in *shc-1* do not alter vulval induction in *let-60(gf)* mutants, indicating that neither FAK nor SHC regulate EGFR signaling during vulval development [41]. Second, integrins form clusters with the EGFR on the plasma membrane to create a micro-environment, in which the EGFR can efficiently interact with EGF ligands and downstream signaling molecules [23,42]. This interaction may involve the induction of EGFR auto-phosphorylation by integrin binding, or signal amplification of EGF-induced receptor activation through integrin-ECM cross-linking [43]. In most of these cases, integrins function as positive regulators of RTK signaling. By contrast, our genetic data indicate that the *C*. *elegans* integrins attenuate EGFR signaling in the vulval cells. RNAi against the talin homolog *tln-1*, *pat-2 α-integrin* or *pat-3 β-integrin* all cause an Apf phenotype characteristic of hyperactive EGFR signaling. Thus, TLN-1 talin is likely to be a critical intracellular mediator of the inhibitory effect, which the integrins exert on the EGFR pathway. The results of the FRAP analysis further indicate that reducing integrin or talin expression causes an increased intracellular mobility and an accelerated recovery of the EGFR on the basolateral plasma membrane. The control of the basolateral EGFR mobility is of particular importance in the vulval cells because the inductive EGF signal is secreted exclusively by the AC, which is located in the somatic gonad facing the basal compartment of the VPCs [32,44]. Hence, only the fraction of EGFR molecules localized on the basolateral plasma membrane can bind EGF and activate RAS/MAPK signaling. We thus propose that the activated αβ*-*integrin/talin complex alters the basolateral membrane micro-environment by recruiting F-actin bundles. Enhanced F-actin recruitment likely reduces the mobility of the EGFR on the basolateral membrane, which in turn inhibits receptor activation and endocytosis [12]. We have previously reported a similar effect on LET-23 EGFR trafficking and signaling in *erm-1(lf)* mutants [34]. The ezrin homolog *erm-1* encodes another FERM domain protein that links F-actin to the basolateral cortex.

Taken together, our results point at a model, in which DEP-1 controls EGFR signaling via two distinct mechanisms (Fig 7). On the one hand, DEP-1 inhibits EGFR signaling by direct de-phosphorylation of the cytoplasmic receptor tail [11,12]. On the other hand, DEP-1 restricts EGFR mobility by regulating the activity of the αβ*-*integrin/talin complex. This dual inhibitory function of DEP-1 ensures a tight regulation of the EGFR signaling pathway. Our findings also provide a good rationale for the reported tumor suppressor activity of DEP-1 in human cancer [6]. Loss of DEP-1 function in cancer cells could simultaneously activate cell proliferation via hyperactive EGFR signaling and increase tumor cell mobility and invasion by reducing integrin-mediated cell adhesion.

## Materials and methods

### *C*. *elegans* methods and strains

The strains used for the experiments were derivates of Bristol strain N2 of *Caenorhabditis elegans*. The animals were cultivated under standard conditions at 20°C as described previously [45]. Unless noted otherwise, the mutations used have been described previously and are listed below by their linkage group. Standard methods were used to construct double and triple mutants. Transgenic animals were generated by microinjection of purified plasmids into the syncytial gonads of young adult worms [46]. All constructs were injected at a concentration of 50 ng/μl (except for the *P*_*bar-1*_::*pat-3(dn)* construct, which was injected at 150ng/μl) with *myo-2*::*mCherry* as transformation marker at a concentration of 2.5 ng/μl or pUnc-119 at 10 ng/μl. The total concentration of DNA was adjusted to 150 ng/μl by adding the plasmid pBluescript-KS. The vulval induction index (VI) was scored at the L4 larval stage using Nomarski optics as described [14]. For Nomarski analysis, animals were mounted on 4% agarose pads in M9 solution containing 20 mM tetramisole hydrochloride.

### Alleles used

LGI: *tln-1(zh117)[gfp*::*tln-1]* (this study). LGII: *dep-1(zh34)* [11], *unc-4(e120)* [45], *rrf-3(pk1426)* [47], *lin-7(e1413)*. LGIII: *unc-119(e2498)*, *unc-119(ed3)*, *unc-119(ed4)* (all [48]), *pat-3(zh105)[pat-3Y792F]*, *pat-3*(*zh115)[pat-3*::*gfp]*, *pat-3*(*zh116)[pat-3Y792F*::*gfp]* (all this study). LGIV: *lip-1(zh15)* [14], *let-60(n1046)* [49]. LGV: *rde-1(ne219)* [50].

Integrated arrays (transgene, cotransformation marker):

LGIV: *zhIs038[let-23*::*GFP; unc-119(+)]*[34]. LG unknown: *qyIs43[pat-3*::*gfp + ina-1(genomic)*, *unc-119(+)]*[27], *qyIs15 [zmp-1>HA-βtail]* [27], *jeIs2222[pat-2*::*gfp*, *rol-6(su1006)]* [26].

Extrachromosomal arrays (transgene, cotransformation marker): *zhEx418[lin-31*::*rde-1*, *myo-2*::*mCherry]* [34], *zhEx419[pat-3*::*gfp Y792A Y804A*, *myo-2*::*mCherry]*, *zhEx420[pat-3*::*gfp Y772A*, *myo-2*::*mCherry]*, *zhEx432[pat-3*::*gfp Y772A Y804A*, *myo-2*::*mCherry]*, *zhEx456[pat-3*::*gfp Y792A*, *myo-2*::*mCherry]*, *zhEx457[pat-3*::*gfp Y804A*, *myo-2*::*mCherry]*, *zhEx458[pat-3*::*gfp TTT796-798AAA*, *myo-2*::*mCherry]*, *zhEx524[pat-3*::*gfp*, *myo-2*::*mCherry]*, *zhEx528[pat-3*::*gfp Y804A*, *myo-2*::*mCherry]* (all this study).

### GST pull-down experiments for LC-MS/MS analyses

The intracellular domain of DEP-1 (wild-type and D1241A) was cloned into the BamHI site of the *E*. *coli* expression vector pGEX-2TK (Pharmacia) as described [11]. Recombinant proteins were affinity-purified on glutathione Sepharose according to the manufacturer’s protocol, except that protein expression was induced in BL21 bacteria at 18°C, and fusion proteins were washed in 20 mM NaP pH = 8.0, 250 mM NaCl, 1% Triton-X. Approximately 50 μg of each DEP-1 fusion protein (wild-type/D1241A) and 100 μg of GST as a negative control were used in each binding reaction. To prepare N2 worm extracts, mixed-stage liquid cultures were cleaned by sucrose flotation, resuspended in lysis buffer (100 mM Tris pH = 8.0, 150 mM NaCl, 1 mM EDTA, 1 mM DTT, 0.5% NP-40, 1x protease inhibitor cocktail; Roche), shock frozen in liquid nitrogen, and homogenized in a swing mill (MM300; Retsch). Thawed worm extract was then centrifuged for 10 min at 4°C and 10,000g to remove insoluble components. About 2.5 mg of total protein extract was used in each reaction. Binding was performed at 4°C overnight, followed by three washes with lysis buffer. Bound proteins were eluted by boiling the beads for 5 min in 30 μl Laemmli buffer and separated on a 4–15% linear gradient SDS-gel (Biorad Nr. 161–1104), followed by colloidal Coomassie blue staining according to the manufacturer’s protocol (Roti-Blue; Roth).

### In-gel digestion and protein identification by LTQ-Orbitrap

Differential protein bands were excised with a scalpel into small pieces and prepared for in-gel tryptic digestion. Thereby the gel pieces were washed and dehydrated three times in 50% Acetonitrile and dried in SpeedVac. 10 mM Dithiothreitol (DTT) in 25 mM Ammonium bicarbonate (AMBIC), pH = 8.0 was added to cover gel pieces and incubated for 45 min at 56°C. After DTT was removed, 50 mM Iodoacetamide (IAM) in AMBIC 25 mM was added to cover gel pieces and incubated for 1 hour at room temperature in the dark. IAM was removed and gel pieces were washed twice in 50% Acetonitrile (ACN) and dried in the SpeedVac. 50 ng trypsin in AMBIC were added for enzymayic digestion and incubated over night at 37°C. To extract the peptides, gel pieces were incubated three times for 15 min with 50% ACN/5% trifluoroacetic acid (TFA) and once with 100% ACN. The peptides were dried in a SpeedVac and resolubilized in 5 μl 3% ACN/0.1% formic acid (FA). Finally, samples were desalted with C18 ZipTip (Millipore) according to the manufacturer’s protocol. Samples were then analyzed on a LTQ-Orbitrap mass spectrometer (Thermo Fischer Scientific, Bremen, Germany) coupled to an Eksigent-Nano-HPLC system (Eksigent Technologies, Dublin (CA), USA). Solvent composition at the two channels was 0.2% formic acid, 1% acetonitrile for channel A and 0.2% formic acid, 80% acetonitrile for channel B. Peptides were resuspended in 3% ACN and 0.2% formic acid and loaded on a self-made tip column (75 μm × 70 mm) packed with reverse phase C18 material (AQ, 3 μm 200 Å, Bischoff GmbH, Leonberg, Germany) and eluted with a flow rate of 200 nl per min by a gradient from 3 to 40% of B in 55 min, 48% B in 60 min, 97% B in 68 min. Full-scan MS spectra (300−2000 m/z) were acquired with a resolution of 60 000 at 400 m/z after accumulation to a target value of 500 000. Collision induced dissociation (CID) MS/MS spectra were recorded in data dependent manner in the ion trap from the five most intense signals above a threshold of 500, using a normalized collision energy of 35% and an activation time of 30 ms. Charge state screening was enabled and singly charge states were rejected. Precursor masses selected twice for MS/MS were excluded for further selection for 120s. The exclusion window was set to 20 ppm, while the size of the exclusion list was set to a maximum of 500 entries. Samples were acquired using internal lock mass calibration set on m/z 429.088735 and 445.120025. MS/MS spectra were exported to Mascot generic format (mgf) and searched against a *C*. *elegans* database (wormbase, April 2010) containing reverse entries and common proteomics contaminants. Mascot search engine version 2.2 was used for protein identification (Perkins et al., 1999), using the following parameters: peptide tolerance at 5 ppm, MS/MS tolerance at 0.8 Da, peptide charge of 2+ or 3+, trypsin as enzyme allowing up to one missed cleavage, carbamidomethylation on cysteines as a fixed modification and oxidation on methionine and phosphorylation of tyrosine as a variable modification. Only peptides with a maximum of 2 (3 for semi-tryptic digest) missed cleavage sites were allowed in database searches. Mascot results (dat files) were compared using Scaffold 3.0 (Proteome Software) filtering the data for proteins identified with at least 2 peptides and a 95% protein Probability.

### GST pull-down experiments for western blot experiments

Approximately 10 μg of purified GST::DEP-1 (wild-type and D1241A) and 40 μg of GST as a negative control were incubated with ca. 800 μg total worm extract over night at 4°C in each binding reaction. Followed by washing with lysis buffer (see above), bound proteins were eluted by boiling the beads for 5 min in Laemmli buffer. GFP tagged proteins were detected on Western blots of 10% acrylamide gels with monoclonal Anti-GFP antibody (Roche, Cat. No. 11 814 460 001). For phosphatase inhibitor experiments, 5 mM Na_3_VO_4_ was added to the lysis buffer.

### CRISPR/Cas9-mediated genome editing

Genome editing was performed using the co-CRISPR strategy described by [51]. For a detailed description of the procedure and the construction of the plasmids used, see S1 Methods.

### RNAi assays

RNAi was performed by feeding worms with dsRNA-producing *E*. *coli* as described [52] with the following modifications: The worms were synchronized with hypochloride solution, and L1 larvae (P0) were placed on growth media plates containing 3 mM IPTG and allowed to grow at 20°C. The F2 generation was analyzed.

### Spinning disc microscopy and FRAP analysis

The PAT-3::GFP and GFP::TLN-1 reporters were imaged with an X-light spinning disc microscope using a 70 μm pinhole, a 475 nm solid state light source (Lumencor Spectra light engine) and a CMOS (Hamamatsu ORCA flash 4.0) or EMCCD (iXon Ultra 888) camera. For each animal, around 50 stacks were taken at 0.3–0.5 μm z-spacing. Images were deconvolved using the Huygens software package (Scientific Volume imaging) and the three mid-sagittal sections around the AC were projected to generate intensity plots with the Fiji software [53] using a custom script described in [35]. For LET-23::GFP FRAP analysis, RNAi treated L3 larvae at the Pn.pxx stage were analyzed using a confocal scanning microscope (Zeiss LSM710 equipped with 458/488/514 nm argon laser) as described [36]. Curve fitting was done using the solver function in MS Excel as described previously [34]. The raw data and data analysis are found in S3 Table.

## Supporting information

S1 Table Numbers of peptides per protein identified by LC-MS/MS analyses in the DEP-1 intra_wt, DEP-1 intra_DA, and GST pull-downs. LC-MS/MS analyses identified 585 proteins. Every pull-down was done in triplicates. The numbers represent the peptides that were detected. Min. Protein Probability = 95%; Min. Number of Peptides = 2. Values from „Protein abundance”correspond to the „*C*. *elegans* PaxDB integrated dataset”(www.pax-db.org).
(PDF)

S2 Table Sequence analysis of predicted off target sites. Potential off-target sites of *pat-3* sgRNA #2 and *pat-3* sgRNA #4, which were rated by the CRISPR Design Tool at www.crispr.mit.edu [29] with an “off-target hit score” ≥0.2, were PCR amplified and sequenced with the indicated forward- and reverse primers. None of the analyzed off-target sites were altered by the CRISPR/CAS9-mediated genome editing. For *tln-1* sgRNA #4 no off-target sites with a score ≥0.2 were predicted.
(PDF)

S3 Table Curve fitting analysis of the LET-23::GFP FRAP data. (XLSX)

S4 Table Sequences of primers used. (PDF)

S1 Fig Coomassie Blue stained SDS-PAGE gels with the purified DEP-1intra-wt and -DA GST fusion proteins used for the pull-down experiment shown in Fig 2D. (TIF)

S1 Methods (DOCX)
