# Supplementary material for: β-Integrin de-phosphorylation by the Density-Enhanced Phosphatase DEP-1 attenuates EGFR signaling in C. elegans
Source: PLoS Genet. 2017 Jan 30;13(1):e1006592. doi: 10.1371/journal.pgen.1006592 (PMC5305270; doi:10.1371/journal.pgen.1006592)
Supplement: S1 Table — LC-MS/MS analyses identified 585 proteins. Every pull-down was done in triplicates. The numbers represent the peptides that were detected. Min. Protein Probability = 95%; Min. Number of Peptides = 2. Values from „Protein abundance”correspond to the „C. elegans PaxDB integrated dataset”(www.pax-db.org). (PDF) [file pgen.1006592.s001.pdf]

S1 Table

Gene name	Accession Number	Brief description	DEP-1 intra wt			DEP-1 intra DA			GST			Protein Abundance (ppm)
pat-2	P34446	Integrin alpha pat-2 (Paralyzed arrest at two-fold protein 2)	4	0	0	43	33	23	0	0	0	58.8
pat-3	Q27874	Integrin beta pat-3 (Paralyzed arrest at two-fold protein 3)	0	0	0	29	18	7	0	0	0	42
nid-1	C7FZU3	Protein F54F3.1b, partially confirmed by transcript evidence	2	2	0	25	16	0	0	0	0	63.8
prp-8	P34369	Pre-mRNA-splicing factor 8 homolog	0	3	0	12	16	0	0	0	0	65.6
rps-6	Q9NEN6	40S ribosomal protein S6	0	10	7	8	15	8	0	0	0	2440
K12D12.1	Q23670	Probable DNA topoisomerase 2	0	2	0	8	9	0	0	0	0	111
puf-12	Q09622	Pumilio domain-containing protein 12	0	0	2	7	5	0	0	0	0	107
vit-4	P18947	Vitellogenin-4	0	0	0	7	4	2	0	0	0	570
apl-1	Q10651	Beta-amyloid-like protein	0	0	0	6	5	0	0	0	0	8.26
F08D12.1	P91240	Signal recognition particle 72 kDa protein homolog	0	0	0	6	0	0	0	0	0	134
rpl-16	Q27389	60S ribosomal protein L13a	9	5	0	5	9	5	0	0	0	2404
eft-1	Q23463	Elongation factor protein 1, confirmed by transcript evidence	0	0	5	5	5	4	0	0	0	70.8
avr-15	Q95PJ6	Protein R11G10.1b, confirmed by transcript evidence	0	0	0	4	2	0	0	0	0	4.21
mcm-5	Q21902	DNA replication licensing factor mcm-5	2	0	2	4	0	3	0	0	0	69.5
C13B9.3	Q09236	Probable coatomer subunit delta (Delta-coat protein)	2	2	0	4	0	0	0	0	0	164
eif-3	O02328	Eukaryotic translation initiation factor 3 subunit C	0	0	0	4	0	0	0	0	0	169
kin-19	P42168	Casein kinase I isoform alpha	0	0	0	4	0	0	0	0	0	160
sym-1	Q93374	Protein C44H4.3	0	0	0	4	0	0	0	0	0	52.2
Q95XR0	Q95XR0	Putative uncharacterized protein	2	0	0	3	5	2	0	0	0	289
pro-3	Q9NEU2	Protein SDA1 homolog	0	0	2	3	3	2	0	0	0	24.6
mrs-1	Q20970	Methionyl-tRNA synthetase, cytoplasmic	0	0	0	3	2	2	0	0	0	120
F58G11.2	P90897	Putative uncharacterized protein F58G11.2	0	0	5	3	0	0	0	0	0	74.9
cnx-1	P34652	Calnexin homolog	0	0	0	3	0	0	0	0	0	72.6
F14B4.3	Q27493	Protein F14B4.3, partially confirmed by transcript evidence	0	0	0	3	0	0	0	0	0	31.7
math-33	O45623	Protein H19N07.2a, confirmed by transcript evidence	0	0	0	3	0	0	0	0	0	71.3
ngp-1	Q56VZ2	Protein T19A6.2c, confirmed by transcript evidence	0	0	0	3	0	0	0	0	0	63.3
T05E11.3	Q22235	Protein T05E11.3a, confirmed by transcript evidence	0	0	0	3	0	0	0	0	0	448
rpl-11.2	Q19162	Ribosomal protein, large subunit protein 11.2	4	4	5	2	5	3	0	0	0	808
car-1	Q9XW17	Protein Y18D10A.17, confirmed by transcript evidence	3	0	3	2	3	3	0	0	0	524
C18B2.3	Q18074	Putative uncharacterized protein	2	3	0	2	3	2	0	0	0	153
rpt-2	O16368	Probable 26S protease regulatory subunit 4	0	2	0	2	2	0	0	0	0	535
K04C2.2	Q21210	Putative uncharacterized protein	0	0	0	2	2	0	0	0	0	65.5
his-24	P10771	Histone H1.1	0	0	0	2	0	2	0	0	0	596
rsp-1	Q23121	Probable splicing factor, arginine/serine-rich 1 (CeSRp75) (RNA-binding protein srp-5)	0	0	2	2	0	0	0	0	0	199
abcf-3	Q20306	Putative uncharacterized protein	0	0	0	2	0	0	0	0	0	96
ama-1	P16356	DNA-directed RNA polymerase II subunit RPB1 (RNA polymerase II subunit B1)	0	0	0	2	0	0	0	0	0	66.1
npp-13	Q9BKT9	Nuclear pore complex protein protein 13, isoform a	0	0	0	2	0	0	0	0	0	102
nsf-1	Q09EE7	Protein H15N14.2b, confirmed by transcript evidence	0	0	0	2	0	0	0	0	0	90.8
nst-1	Q21086	Guanine nucleotide-binding protein-like 3 homolog (Nucleostemin-1)	0	0	0	2	0	0	0	0	0	108
pgl-1	Q304E5	P granule abnormality protein 1, isoform b	0	0	0	2	0	0	0	0	0	97.2
rpb-2	Q10578	RNA polymerase II subunit B2	0	0	0	2	0	0	0	0	0	59.4
tps-2	O45380	Trehalose-6-phosphate synthase 2	0	0	0	2	0	0	0	0	0	27.2
unc-54	Q09981	UNC-45	0	0	0	2	0	0	0	0	0	2658
ZK550.3	O62512	Protein ZK550.3, partially confirmed by transcript evidence	0	0	0	2	0	0	0	0	0	40
Y37E3.8	Q9BKU5	Putative uncharacterized protein	0	4	3	0	5	0	0	0	0	2162
Y62H9A.6	Q9XWT3	Protein Y62H9A.6, confirmed by transcript evidence	0	0	2	0	3	0	0	0	0	565
Y54G2A.2a	Y54G2A.2a	Protein Y54G2A	0	0	0	0	3	0	0	0	0	n.a.
ivd-1	O44446	Isovaleryl-CoA dehydrogenase ivd-1	0	0	0	0	2	2	0	0	0	389
rps-5	P49041	40S ribosomal protein S5	4	4	3	0	2	0	0	0	0	1535
F55A12.5	O01756	Putative uncharacterized protein	3	0	0	0	2	0	0	0	0	28.3
rpl-15	P91374	60S ribosomal protein L15	0	5	0	0	2	0	0	0	0	2131
F44E5.1	Q9XU97	Protein F44E5.1, confirmed by transcript evidence	0	0	0	0	2	0	0	0	0	709
F45H10.2	O45525	Protein F45H10.2, confirmed by transcript evidence	0	0	0	0	2	0	0	0	0	271
F46F11.1	P91309	Inositol hexakisphosphate and diphosphoinositol-pentakisphosphate kinase	0	0	0	0	2	0	0	0	0	18.6
imb-4	Q23089	Importin beta family protein 4, isoform a	0	0	0	0	2	0	0	0	0	46.7
R09F10.8	Q23031	Putative uncharacterized protein	0	0	0	0	2	0	0	0	0	8.03
rpl-38	O17570	60S ribosomal protein L38	0	0	0	0	2	0	0	0	0	1169

plp-1	Q94230	Pur alpha like protein protein 1, confirmed by transcript evidence	0	0	0	0	0	4	0	0	0	468
cey-1	O62213	Protein F33A8.3, confirmed by transcript evidence	0	0	0	0	0	3	0	0	0	1132
K01G5.5	O17919	Putative H/ACA ribonucleoprotein complex subunit 4	0	0	0	0	0	3	0	0	0	245
ret-1	A5JYU4	Protein W06A7.3f	0	0	0	0	0	3	0	0	0	69.1
Y62H9A.3	Q9XWT5	Protein Y62H9A.3, confirmed by transcript evidence	0	0	0	0	0	3	0	0	0	12.9
cdc-48.1	P54811	Transitional endoplasmic reticulum ATPase homolog 1	3	0	3	0	0	2	0	0	0	436
rab-11.1	O01803	Rab family protein 11.1	2	3	0	0	0	2	0	0	0	226
C46G7.2	O02141	Putative uncharacterized protein	0	3	0	0	0	2	0	0	0	211
ran-1	O17915	GTP-binding nuclear protein ran-1 (Ras-related nuclear protein 1)	0	2	0	0	0	2	0	0	0	831
sqd-1	Q8MXR6	Homologous to drosophila sqd (Squid) protein protein 1	0	2	0	0	0	2	0	0	0	1530
C05G5.4	P53596	Succinyl-CoA synthetase subunit alpha	0	0	0	0	0	2	0	0	0	1365
C10G11.7	P91027	Putative uncharacterized protein	0	0	0	0	0	2	0	0	0	547
imp-2	P49049	Intramembrane protease 2	0	0	0	0	0	2	0	0	0	76.7
R04D3.3	Q21713	Protein R04D3.3, partially confirmed by transcript evidence	0	0	0	0	0	2	0	0	0	1.15
rpt-5	O76371	Proteasome regulatory particle, atpase-like protein 5	0	0	0	0	0	2	0	0	0	834
let-711	Q20937	Lethal protein 711, partially confirmed by transcript evidence	2	2	0	0	0	0	0	0	0	26.9
tag-18	Q22508	Temporarily assigned gene name protein 18	2	2	0	0	0	0	0	0	0	131
asp-1	Q9TVS4	Aspartic protease 1	2	0	0	0	0	0	0	0	0	1290
cey-2	P91306	Y-box protein 2, confirmed by transcript evidence	2	0	0	0	0	0	0	0	0	490
cox-2	P24894	Cytochrome c oxidase subunit 2	2	0	0	0	0	0	0	0	0	74.2
D2045.2	Q18983	Protein D2045.2, partially confirmed by transcript evidence	2	0	0	0	0	0	0	0	0	37.2
npp-8	Q95Y15	Nuclear pore complex protein protein 8, isoform b	2	0	0	0	0	0	0	0	0	21.8
pat-4	Q9TZC4	Paralysed arrest at two-fold protein 4, confirmed by transcript evidence	2	0	0	0	0	0	0	0	0	67
R13H4.2	A5Z2W3	Protein R13H4.2a, confirmed by transcript evidence	2	0	0	0	0	0	0	0	0	35.1
rps-30	Q18231	Ribosomal protein, small subunit protein 30, confirmed by transcript evidence	0	2	2	0	0	0	0	0	0	154
larp-1	D5MCN1	Larp (Rna binding la related protein) homolog protein 1	0	2	0	0	0	0	0	0	0	304
dpy-11	Q9UAV4	Dumpy : shorter than wild-type protein 11	0	2	0	0	0	0	0	0	0	135
F25H2.2	Q93566	Protein F25H2.2, confirmed by transcript evidence	0	2	0	0	0	0	0	0	0	1.91
F55C5.8	Q20822	Probable signal recognition particle 68 kDa protein (SRP68)	0	2	0	0	0	0	0	0	0	106
gfi-1	Q94246	Gei-4(Four) interacting protein protein 1	0	2	0	0	0	0	0	0	0	60.9
his-2	P08898	Histone H3	0	2	0	0	0	0	0	0	0	187
ift-74	Q18106	Ift (Chlamydomonas intraflagellar transport) homolog protein 74	0	2	0	0	0	0	0	0	0	< 0.01
K07H8.10	O45181	Putative uncharacterized protein	0	2	0	0	0	0	0	0	0	230
rop-1	Q27274	60 kDa SS-A/Ro ribonucleoprotein homolog	0	2	0	0	0	0	0	0	0	74.8
dhs-3	A5JYX4	Protein T02E1.5a, confirmed by transcript evidence	0	0	2	0	0	0	0	0	0	104
F23B12.4	A8WHS3	Protein F23B12.4a, confirmed by transcript evidence	0	0	2	0	0	0	0	0	0	10.2
kin-3	P18334	Casein kinase II subunit alpha	0	0	2	0	0	0	0	0	0	220
mcm-7	O16297	Yeast mcm (Licensing factor) related protein 7	0	0	2	0	0	0	0	0	0	39.1
tag-174	Q20779	Probable cytochrome c oxidase subunit 6A	0	0	2	0	0	0	0	0	0	379
tba-4	Q20409	Protein F44F4.11, partially confirmed by transcript evidence	0	0	2	0	0	0	0	0	0	426
ubl-1	P37165	Ubiquitin-like protein 1-40S ribosomal protein S27a	0	0	2	0	0	0	0	0	0	592
atp-3	P91283	Atp synthase subunit protein 3, isoform a, confirmed by transcript evidence	6	6	6	0	2	9	0	0	2	1538
trap-3	Q95XS1	Translocon-associated protein protein 3, confirmed by transcript evidence	0	0	0	0	0	0	0	0	2	295
rpl-10	Q09533	60S ribosomal protein L10 (QM protein homolog)	10	12	9	12	11	9	0	2	0	2038
Y25C1A.5	Q9TYL9	Coatomer subunit beta (Beta-coat protein)	0	0	4	7	3	5	0	2	0	127
eif-3.B	Q9XWI6	Eukaryotic translation initiation factor 3 subunit B (eIF3b)	0	0	2	6	0	3	0	2	0	100
egl-45	P34339	Egg-laying defective protein 45 (Eukaryotic translation initiation factor 3 subunit 10)	2	2	0	4	0	3	0	2	0	208
alg-1	B6VQ75	Protein F48F7.1b	0	0	4	3	2	2	0	2	0	72.5
Y59A8A.3	Q9GRZ9	Protein Y59A8A.3, confirmed by transcript evidence	0	0	2	3	0	4	0	2	0	362
rps-24	Q1XFY9	Ribosomal protein, small subunit protein 24	0	3	6	2	4	5	0	2	0	2060
lmp-1	Q11117	LAMP family protein lmp-1	2	2	2	2	0	0	0	2	0	139
imb-5	Q965V4	Importin beta family protein 5	0	0	4	2	0	0	0	2	0	45.7
dnj-29	Q9U1V9	Protein Y63D3A.6b, confirmed by transcript evidence	0	0	0	2	0	0	0	2	0	73.6
npp-9	Q21021	Protein F59A2.1a, confirmed by transcript evidence	0	0	0	2	0	0	0	2	0	217
rskn-2	Q18846	PRibosomal protein S6 kinase alpha-5 homolog	0	0	0	2	0	0	0	2	0	45.8
spl-1	Q9Y194	Sphingosine-1-phosphate aldolase	0	0	0	2	0	0	0	2	0	95.9
rpl-32	Q22716	Protein T24B8.1a, confirmed by transcript evidence	2	0	2	0	3	3	0	2	0	2363
rps-27	Q9TXP0	40S ribosomal protein S27	0	0	0	0	2	2	0	2	0	191
unc-116	P34540	Kinesin heavy chain (Uncoordinated protein 116)	0	3	2	0	2	0	0	2	0	179
rpl-25.1	P48162	60S ribosomal protein L23a 1	0	0	0	0	2	0	0	2	0	1316
tba-3	P91910	Tubulin alpha-3 chain (Mechanosensory abnormality protein 12)	0	0	0	0	2	0	0	2	0	
prmt-1	Q9U2X0	Protein Y113G7B.17, confirmed by transcript evidence	2	0	0	0	0	0	0	2	0	446
Y57G11C	O18239	Putative uncharacterized protein Y57G11C.15	2	0	0	0	0	0	0	2	0	190
nuo-1	Q17880	Protein C09H10.3, confirmed by transcript evidence	0	2	2	0	0	0	0	2	0	249
gst-16	Q93698	Protein F37B1.5, partially confirmed by transcript evidence	0	2	0	0	0	0	0	2	0	24.4
rla-2	Q9U1X9	Protein Y62E10A.1, confirmed by transcript evidence	0	0	2	0	0	0	0	2	0	5637
Y67H2A.5	Q95PZ1	Protein Y67H2A.5, confirmed by transcript evidence	0	0	2	0	0	0	0	2	0	670

acdh-11	Q3T978	Protein Y45F3A.3b, confirmed by transcript evidence	0	0	0	0	0	0	0	2	0	68.6
acs-11	Q20264	Fatty acid coa synthetase family protein 11, partially confirmed by transcript evidence	0	0	0	0	0	0	0	2	0	294
acs-5	Q9XWD1	Protein Y76A2B.3, confirmed by transcript evidence	0	0	0	0	0	0	0	2	0	83.7
arp-1	Q9NA98	Protein Y53F4B.22, confirmed by transcript evidence	0	0	0	0	0	0	0	2	0	202
C34F11.3	D7SFL2	Putative uncharacterized protein	0	0	0	0	0	0	0	2	0	170
clp-1	P34308	Calpain clp-1	0	0	0	0	0	0	0	2	0	203
D1005.1	P53585	Probable ATP-citrate synthase	0	0	0	0	0	0	0	2	0	152
dpf-3	O44987	Dipeptidyl peptidase four (Iv) family protein 3	0	0	0	0	0	0	0	2	0	40.1
emb-8	Q09590	NADPH--cytochrome P450 reductase	0	0	0	0	0	0	0	2	0	75.9
F07A11.2	Q19130	Protein F07A11.2a, confirmed by transcript evidence	0	0	0	0	0	0	0	2	0	22.6
gsk-3	Q9U2Q9	Glycogen synthase kinase-3	0	0	0	0	0	0	0	2	0	177
gsnl-1	Q21253	Gelsolin-like protein 1	0	0	0	0	0	0	0	2	0	136
gsp-1	Q27497	Serine/threonine-protein phosphatase PP1-alpha	0	0	0	0	0	0	0	2	0	202
H03A11.2	Q9XTW1	Protein H03A11.2, partially confirmed by transcript evidence	0	0	0	0	0	0	0	2	0	48.1
haf-4	Q9TZD9	Half transporter (Pgp related) protein 4	0	0	0	0	0	0	0	2	0	56.1
hpo-29	Q8WTL6	Putative uncharacterized protein	0	0	0	0	0	0	0	2	0	n.a.
immt-1	Q22505	Putative uncharacterized protein	0	0	0	0	0	0	0	2	0	294
nduf-5	Q9N3D9	Putative uncharacterized protein	0	0	0	0	0	0	0	2	0	453
npl-4.2	O01894	Putative uncharacterized protein	0	0	0	0	0	0	0	2	0	37.2
pod-2	Q9GZI3	Polarity and osmotic sensitivity defect protein 2, isoform a	0	0	0	0	0	0	0	2	0	57.6
ppw-1	O02095	Paz/piwi domain-containing protein 1	0	0	0	0	0	0	0	2	0	34.9
ppw-2	Q9N585	Paz/piwi domain-containing protein 2	0	0	0	0	0	0	0	2	0	29.4
R02D3.1	O44503	Putative uncharacterized protein R02D3.1	0	0	0	0	0	0	0	2	0	179
ran-3	Q18211	Regulator of chromosome condensation (RCC1 homolog)	0	0	0	0	0	0	0	2	0	182
rpn-5	Q19324	Proteasome regulatory particle, non-atpase-like protein 5	0	0	0	0	0	0	0	2	0	223
tag-320	Q11067	Probable protein disulfide-isomerase A6	0	0	0	0	0	0	0	2	0	415
tba-1	O18688	Protein F26E4.8, confirmed by transcript evidence	0	0	0	0	0	0	0	2	0	573
ttr-2	P34500	Transthyretin-like protein 2	0	0	0	0	0	0	0	2	0	364
ttr-24	Q9XXR4	Protein Y51A2D.9, confirmed by transcript evidence	0	0	0	0	0	0	0	2	0	119
Y71H10B.1	Q86MI3	Putative uncharacterized protein	0	0	0	0	0	0	0	2	0	74
Y71H2AR.1	Q9BL27	Putative uncharacterized protein	0	0	0	0	0	0	0	2	0	184
ZK669.4	Q23571	Protein ZK669.4, confirmed by transcript evidence	0	0	0	0	0	0	0	2	0	174
ZK829.7	Q23624	Protein ZK829.7, confirmed by transcript evidence	0	0	0	0	0	0	0	2	0	175
ZK836.2	Q23629	Probable 2-oxoglutarate dehydrogenase E1 component DHKTD1 homolog	0	0	0	0	0	0	0	2	0	117
cyp-25a5	O44485	Cytochrome p450 family protein 25A5	0	0	0	0	0	0	0	2	0	n.a.
rps-25	P52821	40S ribosomal protein S25	2	2	6	2	5	6	0	2	2	2175
B0303.3	P34255	Uncharacterized protein B0303.3	0	0	3	2	4	3	0	2	2	426
rpl-31	Q9U332	60S ribosomal protein L31	0	0	3	0	2	2	0	2	2	864
nduf-7	Q94360	NADH-ubiquinone oxidoreductase 20 kDa subunit	0	0	0	0	0	0	0	2	2	n.a.
Y71F9AL.17	Q9N4H7	Putative uncharacterized protein	4	0	0	10	4	5	0	3	0	145
T22D1.4	Q9GZH4	Putative uncharacterized protein	0	3	2	2	0	0	0	3	0	198
rpl-35	P34662	60S ribosomal protein L35	0	3	3	0	2	2	0	3	0	4176
let-805	Q9UB28	Myotactin form B	0	0	0	0	2	0	0	3	0	87.1
alh-12	Q7Z1Q2	Aldehyde dehydrogenase protein 12, isoform b	0	0	0	0	0	0	0	3	0	391
apb-1	Q9N4F3	APB-1 protein, isoform a	0	0	0	0	0	0	0	3	0	122
C32F10.8	O01685	Putative uncharacterized protein C32F10.8	0	0	0	0	0	0	0	3	0	417
C41G7.9	B3WFW9	Protein C41G7.9a, confirmed by transcript evidence	0	0	0	0	0	0	0	3	0	277
drp-1	Q8WQC9	Dynamin related protein protein 1, isoform b	0	0	0	0	0	0	0	3	0	76.2
F42G9.1	P49595	Probable protein phosphatase 2C F42G9.1	0	0	0	0	0	0	0	3	0	137
F52E4.5	Q20675	Putative uncharacterized protein	0	0	0	0	0	0	0	3	0	131
glh-1	P34689	ATP-dependent RNA helicase glh-1	0	0	0	0	0	0	0	3	0	119
H19N07.1	O45622	Protein H19N07.1a, confirmed by transcript evidence	0	0	0	0	0	0	0	3	0	176
haf-9	O44897	Half transporter (Pgp related) protein 9, isoform a	0	0	0	0	0	0	0	3	0	74.2
M106.4	Q09580	Glutamine amidotransferase	0	0	0	0	0	0	0	3	0	98.7
mvk-1	Q9N4Z7	Putative uncharacterized protein	0	0	0	0	0	0	0	3	0	n.a.
oig-2	Q9XWM1	Protein Y38F1A.9, partially confirmed by transcript evidence	0	0	0	0	0	0	0	3	0	179
pdi-3	O17908	CeERp57	0	0	0	0	0	0	0	3	0	1362
R06C7.1	Q21770	Germ cell-expressed protein R06C7.1	0	0	0	0	0	0	0	3	0	42.2
rnr-1	Q03604	Ribonucleotide reductase large subunit	0	0	0	0	0	0	0	3	0	69.1
rps-21	P49197	40S ribosomal protein S21	0	0	0	0	0	0	0	3	0	2616
T20B3.1	Q9XUN8	Protein T20B3.1, confirmed by transcript evidence	0	0	0	0	0	0	0	3	0	49.2
tax-6	Q0G819	Protein C02F4.2c, partially confirmed by transcript evidence	0	0	0	0	0	0	0	3	0	101
Y67D2.3	Q9BKQ9	Putative uncharacterized protein	0	0	0	0	0	0	0	3	0	262
fbp-1	Q9N2M2	Fructose-1,6-biphosphatase protein 1	3	0	4	2	0	3	0	3	2	690
rpn-3	Q04908	26S proteasome regulatory subunit rpn-3	0	0	0	2	0	2	0	3	2	306
F38E11.5	Q20168	Probable coatomer subunit beta' (Beta'-coat protein))	2	0	3	6	0	5	0	3	3	217
F59C6.5	Q93831	Protein F59C6.5, confirmed by transcript evidence	0	0	0	0	0	0	0	3	3	405

vit-1	P55155	Vitellogenin-1	7	4	7	39	30	13	0	4	0	715
abcf-2	Q9XTD9	Protein T27E9.7, confirmed by transcript evidence	2	2	0	3	0	0	0	4	0	112
Y46G5A.4	Q9U2G0	Putative U5 small nuclear ribonucleoprotein 200 kDa helicase (EC 3.6.4.13)	0	5	0	2	3	0	0	4	0	56.4
ears-1	Q23315	Protein ZC434.5, confirmed by transcript evidence	2	0	0	2	2	3	0	4	0	
pyr-1	Q18990	Protein D2085.1, partially confirmed by transcript evidence	0	2	0	2	0	0	0	4	0	103
crt-1	P27798	Calreticulin	0	2	0	0	2	0	0	4	0	3113
nmt-1	P46548	Myristoyl-CoA:protein N-myristoyltransferase	2	3	0	0	0	0	0	4	0	163
msp-10	P05634	Major sperm protein 10/36/56/76 (MSP)	0	2	2	0	0	0	0	4	0	56.8
rpl-36	P49181	60S ribosomal protein L36	0	0	3	0	0	0	0	4	0	1493
cyn-15	Q9U1Q3	Protein Y87G2A.6, confirmed by transcript evidence	0	0	0	0	0	0	0	4	0	47.1
F55H12.4	P90889	Protein F55H12.4, confirmed by transcript evidence	0	0	0	0	0	0	0	4	0	72.9
iff-2	Q20751	Eukaryotic translation initiation factor 5A-2	0	0	0	0	0	0	0	4	0	643
lrp-1	Q04833	Low-density lipoprotein receptor-related protein (LRP)	0	0	0	0	0	0	0	4	0	16.1
T04A8.7	Q22137	Protein T04A8.7a, confirmed by transcript evidence	0	0	0	0	0	0	0	4	0	143
ZC416.6	O44183	Putative uncharacterized protein ZC416.6	0	0	0	0	0	0	0	4	0	16.5
egl-4	O76360	cGMP-dependent protein kinase egl-4	3	2	3	8	4	2	0	5	0	113
ars-2	O01541	Alanyl-tRNA synthetase, cytoplasmic	2	2	2	5	2	0	0	5	0	292
alh-3	Q19428	Protein F36H1.6, confirmed by transcript evidence	0	0	0	4	0	0	0	5	0	412
R05F9.6	Q21742	Putative uncharacterized protein	4	3	0	3	0	2	0	5	0	378
ddb-1	Q21554	DNA damage-binding protein 1	2	0	0	0	2	0	0	5	0	32.1
grs-1	Q10039	Glycyl-tRNA synthetase	0	2	0	0	0	0	0	5	0	344
fars-1	Q86B36	Phenylalanyl amino-acyl trna synthetase protein 1, isoform b	0	0	0	0	0	0	0	5	0	
pars-1	Q22620	Prolyl trna synthetase protein 1, isoform a	0	0	0	0	0	0	0	5	0	
spc-1	Q21408	Spectrin protein 1	0	0	0	0	0	0	0	5	0	295
unc-18	P34815	Putative acetylcholine regulator unc-18	0	0	0	0	0	0	0	5	0	65
unc-22	D3YT57	unc-22	0	0	0	0	0	0	0	5	0	132
unc-70	E0AHA7	Uncoordinated protein 70, isoform c	0	0	0	0	0	0	0	5	0	181
unc-89	O01761	Muscle M-line assembly protein unc-89	0	0	0	0	0	0	0	5	0	64.7
W09C5.8	Q9U329	Protein W09C5.8, confirmed by transcript evidence	0	0	2	0	0	0	0	5	2	354
uba-1	C1P636	Protein C47E12.5c, confirmed by transcript evidence	5	5	0	4	0	2	0	6	0	335
eel-1	Q9GUP2	Enhancer of efl-1 mutant phenotype protein 1	0	0	0	0	0	0	0	6	0	42.1
gspd-1	Q27464	Glucose-6-phosphate 1-dehydrogenase	0	0	0	0	0	0	0	6	0	123
vrs-2	Q9U1Q4	Valyl-tRNA synthetase	4	0	3	8	0	2	0	7	0	209
tsn-1	Q19328	Tudor staphylococcal nuclease homolog protein 1	4	0	6	4	2	7	0	7	0	697
gsy-1	Q9U2D9	Probable glycogen [starch] synthase	0	2	0	4	0	0	0	7	0	223
lev-11	Q22866	Tropomyosin isoforms a/b/d/f (Levamisole resistant protein 11)	13	7	0	7	0	4	0	8	8	7575
npp-12	P91495	Nuclear pore complex protein protein 12	2	0	0	0	0	0	0	10	0	51.6
ketn-1	A7DT47	Kettin (Drosophila actin-binding) homolog protein 1	0	0	0	0	0	0	0	13	0	134
nmy-1	Q20641	Non-muscle myosin protein 1	0	0	0	2	0	0	0	17	2	114
fasn-1	P91871	Protein F32H2.5, partially confirmed by transcript evidence	9	7	0	12	8	0	0	21	0	147
dhc-1	Q19020	Dynein heavy chain, cytoplasmic (Dynein heavy chain, cytosolic) (DYHC)	0	8	0	0	4	0	0	29	0	65.1
gcn-1	B3CJ34	Putative uncharacterized protein	16	14	0	20	21	0	0	42	0	75.2
T08G11.1	Q8T3D2	Protein T08G11.1b, partially confirmed by transcript evidence	0	2	0	0	0	0	0	55	0	69.1
clu-1	P34466	Protein KIAA0664 homolog	2	3	3	7	4	5	2	0	0	116
F54F11.2	B6VQ96	Protein F54F11.2b, partially confirmed by transcript evidence	0	0	0	3	0	0	2	0	0	127
got-2	Q17994	Aspartate aminotransferase	2	0	2	2	0	0	2	0	0	
asp-4	Q21966	Protein R12H7.2, confirmed by transcript evidence	2	0	0	2	0	0	2	0	0	588
tag-241	D3KFS7	Protein C34E11.3a	0	0	0	2	0	0	2	0	0	
rpl-37a	Q9U2A8	60S ribosomal protein L37a	0	0	3	0	3	0	2	0	0	83.8
rpl-26	Q19869	60S ribosomal protein L26	0	5	3	0	2	0	2	0	0	1712
aldo-2	P46563	Fructose-bisphosphate aldolase 2	2	0	0	0	0	2	2	0	0	2697
Y53G8AL.2	Q9N3H3	Putative uncharacterized protein	0	0	0	0	0	2	2	0	0	285
F44G3.2	O45518	Protein F44G3.2, confirmed by transcript evidence	5	0	4	0	0	0	2	0	0	96.6
goa-1	P51875	Guanine nucleotide-binding protein G(o) subunit alpha	2	0	2	0	0	0	2	0	0	115
F23B12.5	Q19749	Pyruvate dehydrogenase complex component E2	2	0	0	0	0	0	2	0	0	593
sdz-8	P90780	Protein C55A6.5, partially confirmed by transcript evidence	2	0	0	0	0	0	2	0	0	144
sod-2	P31161	Superoxide dismutase	0	3	0	0	0	0	2	0	0	210
R09B3.3	O45713	Protein R09B3.3, confirmed by transcript evidence	0	0	2	0	0	0	2	0	0	5025
bre-1	Q18801	GDP-mannose 4,6 dehydratase 1	0	0	0	0	0	0	2	0	0	2.11
C24A3.2	Q18124	Putative uncharacterized protein	0	0	0	0	0	0	2	0	0	35.1
cyn-7	P52015	Peptidyl-prolyl cis-trans isomerase 7	0	0	0	0	0	0	2	0	0	2407
cyt-1	P41956	Succinate dehydrogenase cytochrome b560 subunit, mitochondrial	0	0	0	0	0	0	2	0	0	
eif-3	O61820	Eukaryotic translation initiation factor 3 subunit E	0	0	0	0	0	0	2	0	0	174
F32A7.5	P91859	Protein F32A7.5a, partially confirmed by transcript evidence	0	0	0	0	0	0	2	0	0	229
let-70	P35129	Ubiquitin-conjugating enzyme E2 2	0	0	0	0	0	0	2	0	0	332
lpd-9	D5MCR9	Protein T21C9.5b, confirmed by transcript evidence	0	0	0	0	0	0	2	0	0	123
ndk-1	Q93576	Protein F25H2.5, confirmed by transcript evidence	0	0	0	0	0	0	2	0	0	

srs-2	Q18678	Serine--tRNA ligase	0	0	0	0	0	0	2	0	0	300
T08H10.1	Q22352	Putative uncharacterized protein T08H10.1	0	0	0	0	0	0	2	0	0	296
ttr-41	Q86NH9	Putative uncharacterized protein	0	0	0	0	0	0	2	0	0	281
ubc-9	Q95017	Ubiquitin-conjugating enzyme E2 9	0	0	0	0	0	0	2	0	0	142
W09H1.5	O45903	Probable trans-2-enoyl-CoA reductase 1	0	0	0	0	0	0	2	0	0	71
rpl-17	Q9BL19	60S ribosomal protein L17	0	5	4	0	4	7	2	0	2	1932
hpo-18	O16298	Putative uncharacterized protein	0	0	0	0	2	0	2	0	2	n.a.
pmt-2	Q22993	Putative uncharacterized protein	0	0	0	0	0	0	2	0	2	556
nra-4	Q8ITW0	Putative uncharacterized protein	2	3	0	5	0	2	2	2	0	n.a.
lrs-1	Q09996	Leucyl-tRNA synthetase	0	3	0	3	0	2	2	2	0	161
csq-1	Q20203	Protein F40E10.3, confirmed by transcript evidence	0	0	0	2	0	0	2	2	0	250
cpl-1	O45734	Protein T03E6.7, confirmed by transcript evidence	2	4	0	0	3	5	2	2	0	589
ril-1	O17694	Protein C53A5.1, confirmed by transcript evidence	0	0	0	0	3	0	2	2	0	437
ZK1073.1	O02485	Uncharacterized protein ZK1073.1	0	0	0	0	0	2	2	2	0	212
his-11	P04255	Histone H2B 1	0	2	0	0	0	0	2	2	0	426
nrs-1	Q19722	Asparaginyl-tRNA synthetase	0	2	0	0	0	0	2	2	0	311
cct-5	P47209	T-complex protein 1 subunit epsilon	0	0	0	0	0	0	2	2	0	723
F45H10.3	O02267	Protein F45H10.3, confirmed by transcript evidence	0	0	0	0	0	0	2	2	0	436
hsp-4	P20163	Heat shock 70 kDa protein D	0	0	0	0	0	0	2	2	0	440
rrt-1	Q19825	Probable arginyl-tRNA synthetase	0	0	0	0	0	0	2	2	0	146
spg-7	Q9N3T5	Spg (Spastic paraplegia) protein 7	0	0	0	0	0	0	2	2	0	115
T20B12.7	P41847	Anamorsin homolog (Fe-S cluster assembly protein DRE2 homolog)	0	0	0	0	0	0	2	2	0	379
Y39B6A.3	A5HWB2	Protein Y39B6A.3b, confirmed by transcript evidence	0	0	0	0	0	0	2	2	0	9.24
T14G10.5	Q22498	Probable coatomer subunit gamma	4	4	2	4	3	4	2	2	2	158
F43E2.7	O02093	Putative uncharacterized protein F43E2.7	2	0	3	0	0	2	2	2	2	255
R53.4	Q22021	Putative ATP synthase subunit f, mitochondrial	0	3	3	0	3	2	2	2	3	386
mmcm-1	Q23381	MethylmalonylCoA mutase homolog 1	0	0	0	3	0	0	2	3	0	205
krs-1	Q22099	Lysyl-tRNA synthetase	0	0	0	2	0	0	2	3	0	280
par-5	P41932	14-3-3-like protein 1 (Partitioning defective protein 5)	3	3	0	0	0	2	2	3	0	1754
C30F12.7	Q95YD8	Putative uncharacterized protein	3	0	0	0	0	0	2	3	0	175
rpa-2	O01504	60S acidic ribosomal protein P2	2	0	4	0	0	0	2	3	0	138
paa-1	Q09543	Protein phosphatase PP2A regulatory subunit A	0	4	0	0	0	0	2	3	0	432
K02F3.2	Q21153	Probable calcium-binding mitochondrial carrier K02F3.2	0	0	0	0	0	0	2	3	0	138
sqv-4	Q19905	UDP-glucose 6-dehydrogenase (Squashed vulva protein 4)	0	0	0	0	0	0	2	3	0	181
trap-4	Q9U238	Protein Y56A3A.21, confirmed by transcript evidence	0	0	0	0	0	0	2	3	0	360
trs-1	P52709	Threonyl-tRNA synthetase	0	0	0	0	0	0	2	3	0	165
F36A2.7	P90860	Protein F36A2.7, confirmed by transcript evidence	0	0	0	0	2	2	2	3	2	1020
asb-1	Q20053	Protein F35G12.10, confirmed by transcript evidence	0	3	0	0	0	2	2	3	2	138
vit-3	Q9N4J2	Vitellogenin-3	7	3	2	25	17	5	2	4	0	595
K07C5.4	Q21276	Uncharacterized NOP5 family protein K07C5.4	5	5	10	15	12	11	2	4	0	411
alh-13	P54889	Aldehyde dehydrogenase family 13	0	0	3	4	0	0	2	4	0	104
rpl-27	P91914	60S ribosomal protein L27	0	4	8	2	5	5	2	4	0	2873
ads-1	O45218	Alkyldihydroxyacetonephosphate synthase	2	0	2	0	0	0	2	4	0	128
R05H10.5	O62327	Probable glutathione peroxidase	0	0	0	0	0	0	2	4	0	236
hrp-2	Q9NLD1	Protein F58D5.1a, confirmed by transcript evidence	3	5	0	3	0	0	2	4	2	386
pab-1	Q9U302	Protein Y106G6H.2a, confirmed by transcript evidence	13	13	2	13	0	3	2	5	0	840
laf-1	Q4W5R4	Lethal and feminizing protein 1, confirmed by transcript evidence	8	15	2	12	0	4	2	5	0	171
cpt-1	Q9U2F2	Protein Y46G5A.17, confirmed by transcript evidence	0	0	6	6	0	2	2	5	0	51.3
rps-13	P51404	40S ribosomal protein S13	2	6	2	4	6	2	2	5	0	2561
gst-29	Q9NAB1	Protein Y53F4B.32, confirmed by transcript evidence	6	8	2	2	3	2	2	5	0	26.2
F58F9.7	Q20992	Acyl-coenzyme A oxidase	0	0	0	0	0	0	2	5	0	84.2
rps-17	O01692	40S ribosomal protein S17	2	4	4	0	4	3	2	5	2	3320
lec-3	Q09581	32 kDa beta-galactoside-binding lectin lec-3	0	0	0	0	0	0	2	6	0	419
nex-2	Q27512	Protein T07C4.9a, confirmed by transcript evidence	0	0	0	0	0	0	2	6	0	64.8
cgh-1	Q95YF3	ATP-dependent RNA helicase cgh-1	4	6	5	5	3	5	2	6	2	265
ers-1	O62431	Probable glutaminyl-tRNA synthetase	2	2	3	8	4	4	2	7	0	213
nol-5	O45012	Putative uncharacterized protein	2	4	8	5	2	8	2	7	0	353
C14C10.5	Q17971	Protein C14C10.5, partially confirmed by transcript evidence	0	0	0	0	0	0	2	7	0	42.5
cpt-2	Q17831	Protein R07H5.2a, confirmed by transcript evidence	0	0	0	0	0	0	2	7	0	136
pdi-2	Q17770	Protein disulfide-isomerase 2	0	0	0	0	0	0	2	7	0	3642
W06H3.3	Q9XXN1	Protein W06H3.3, partially confirmed by transcript evidence	0	0	2	3	0	0	2	8	0	119
frs-2	Q19713	Phenylalanyl-tRNA synthetase beta chain	2	2	0	0	0	0	2	8	0	181
F20D6.11	Q19655	Putative uncharacterized protein	0	0	0	0	0	0	2	8	0	130
T23E7.2	O17338	Putative uncharacterized protein	3	4	0	9	4	2	3	0	0	397
cpn-3	O01542	Calponin protein 3	2	2	3	0	2	2	3	0	0	2021
F27D4.1	Q93615	Probable electron transfer flavoprotein subunit alpha	0	0	0	0	0	3	3	0	0	903
B0250.5	Q9XTI0	Probable 3-hydroxyisobutyrate dehydrogenase	0	0	0	0	0	2	3	0	0	262

mdt-28	A8WHP8	Protein W01A8.1c, confirmed by transcript evidence	0	0	2	0	0	0	3	0	0	258
C50F7.4	P53589	Succinyl-CoA synthetase beta chain	0	0	0	0	0	0	3	0	0	246
cpz-1	O01850	Cathepsin Z-like enzyme cpz-1	0	0	0	0	0	0	3	0	0	189
cts-1	P34575	Probable citrate synthase	0	0	0	0	0	0	3	0	0	1582
F32D1.5	O16294	Guanosine 5'-monophosphate oxidoreductase	0	0	0	0	0	0	3	0	0	434
iff-1	P34563	Eukaryotic translation initiation factor 5A-1	0	0	0	0	0	0	3	0	0	882
pfn-1	Q9XW16	Profilin-1	0	0	0	0	0	0	3	0	0	804
vha-14	P34462	V-type proton ATPase subunit D	0	0	0	0	0	0	3	0	0	476
rpl-10a	Q9N4I4	60S ribosomal protein L10a	3	4	4	6	4	6	3	0	2	2038
rpl-20	O44480	60S ribosomal protein L18a	0	7	4	4	3	7	3	0	2	2610
acdh-3	O44549	Putative uncharacterized protein	5	2	3	4	0	3	3	2	0	705
his-1	P62784	Histone H4	0	2	3	2	2	0	3	2	0	681
rpt-3	P46502	Probable 26S protease regulatory subunit 6B	0	0	0	2	0	2	3	2	0	370
gst-23	P91505	Glutathione s-transferase protein 23, partially confirmed by transcript evidence	2	2	0	2	0	0	3	2	0	NA
rpl-25	Q20647	60S ribosomal protein L23a 2	0	6	4	0	4	3	3	2	0	2133
rpl-28	Q21930	60S ribosomal protein L28	3	2	3	0	3	2	3	2	0	2695
rps-28	Q95Y04	40S ribosomal protein S28	0	2	0	0	3	0	3	2	0	2729
gst-24	Q93694	Protein F37B1.1, confirmed by transcript evidence	0	4	0	0	2	0	3	2	0	47.2
rpa-1	P91913	60S acidic ribosomal protein P1 (Ribosomal protein large subunit P1)	3	0	0	0	0	3	3	2	0	139
gpdh-2	A7LPE6	Protein K11H3.1d, confirmed by transcript evidence	0	0	0	0	0	3	3	2	0	474
unc-87	P37806	Protein unc-87 (Uncoordinated protein 87)	0	2	0	0	0	2	3	2	0	626
hgo-1	Q9Y041	Homogentisic acid oxidase	0	0	0	0	0	0	3	2	0	222
T12D8.10	Q69ZI6	Protein T12D8.10, confirmed by transcript evidence	0	0	0	0	0	0	3	2	0	263
gpb-1	P17343	Guanine nucleotide-binding protein subunit beta-1	0	0	0	0	0	2	3	2	2	345
acdh-10	Q22347	Probable medium-chain specific acyl-CoA dehydrogenase 10,	5	0	2	4	0	0	3	2	3	451
rpt-6	Q9XTT9	Protein Y49E10.1, confirmed by transcript evidence	0	0	0	2	2	0	3	3	0	282
R04F11.2	Q21732	Protein R04F11.2, confirmed by transcript evidence	0	0	4	0	2	2	3	3	0	1053
eif-3	Q95QW0	Eukaryotic translation initiation factor 3 subunit L (eIF3l)	2	0	2	0	0	0	3	3	0	
rme-1	Q86S80	Receptor mediated endocytosis protein 1, isoform f	2	0	0	0	0	0	3	3	0	182
mbf-1	Q9XTV4	Protein H21P03.1, confirmed by transcript evidence	0	0	0	0	0	0	3	3	0	432
Y38F2AR.9	Q95XS2	Putative uncharacterized protein	0	0	0	0	0	0	3	3	0	53.5
rps-23	Q19877	40S ribosomal protein S23	2	2	4	3	5	4	3	3	2	2491
T09A5.11	P45971	Oligosaccharyl transferase 48 kDa subunit	3	3	4	2	2	3	3	3	2	177
rps-22	O17218	Ribosomal protein, small subunit protein 22, isoform a,	2	4	10	0	6	3	3	3	2	2759
rpl-23	P48158	60S ribosomal protein L23	0	2	6	2	4	4	3	3	3	2135
cand-1	Q9XTJ0	Protein Y102A5A.1, partially confirmed by transcript evidence	5	4	4	5	3	3	3	4	0	123
tag-203	O44985	Temporarily assigned gene name protein 203	0	0	0	3	0	0	3	4	0	75.7
ucr-2.2	Q22370	Protein T10B10.2, confirmed by transcript evidence	0	0	0	0	0	0	3	4	0	472
rpl-13	P91128	60S ribosomal protein L13	8	10	3	4	7	4	3	4	2	2209
rps-10	O01869	Ribosomal protein, small subunit protein 10	4	4	5	2	2	3	3	4	3	2017
alh-8	P52713	Malonate-semialdehyde dehydrogenase	2	4	2	3	2	3	3	5	0	1955
rpl-3	P50880	60S ribosomal protein L3	11	15	5	13	15	4	3	5	2	2393
sip-1	Q20363	Stress-induced protein 1	0	2	0	0	0	3	3	5	3	2326
drs-1	Q03577	Aspartyl-tRNA synthetase	5	0	6	4	2	6	3	6	0	255
cct-4	P47208	T-complex protein 1 subunit delta	0	0	0	0	0	0	3	6	0	857
acs-4	Q20121	Fatty acid coa synthetase family protein 4, confirmed by transcript evidence	4	2	4	9	0	5	3	6	2	148
cct-7	Q9TZS5	Chaperonin containing tcp-1 protein 7, isoform a, confirmed by transcript evidence	2	2	2	0	0	3	3	6	2	476
C28H8.3	Q09475	Uncharacterized helicase C28H8.3	3	2	3	6	0	2	3	7	0	87.2
R12C12.1	Q21962	Putative uncharacterized protein	0	0	3	5	3	2	3	7	0	210
sdhb-1	Q09545	Succinate dehydrogenase	0	3	0	0	0	0	3	7	0	340
R05D3.9	P34542	Uncharacterized protein R05D3.9	0	0	0	0	0	0	3	7	0	52.6
rps-18	O18240	Protein Y57G11C.16, confirmed by transcript evidence	2	7	6	3	6	4	3	7	3	4920
rpl-22	P52819	60S ribosomal protein L22	0	7	13	7	11	8	3	8	2	3299
rpn-1	Q9GZH5	Proteasome regulatory particle, non-atpase-like protein 1	3	2	9	11	5	5	3	9	5	255
nuo-5	Q9N4Y8	Nadh ubiquinone oxidoreductase protein 5, isoform a	2	4	5	10	2	7	3	12	3	540
W07E11.1	Q22275	Protein W07E11.1, partially confirmed by transcript evidence	2	5	0	0	0	0	3	18	2	111
myo-5	Q21000	Putative uncharacterized protein	4	3	0	3	0	0	3	19	2	141
vha-8	Q95X44	Vacuolar h atpase protein 8	3	4	0	3	0	3	4	0	0	1473
fmo-2	Q21310	Flavin monooxygenase	2	0	0	2	0	2	4	0	0	3.83
rpn-6	Q20938	Probable 26S proteasome regulatory subunit rpn-6.1	0	0	2	2	0	0	4	0	0	302
rpt-4	O17071	Proteasome regulatory particle ATPase-like protein 4	0	0	2	0	2	0	4	0	0	494
rpt-1	Q18787	Proteasome 26S subunit ATPase 2	0	0	0	0	0	0	4	0	0	322
C04C3.3	O44451	Pyruvate dehydrogenase E1 component subunit beta	3	2	0	4	0	3	4	0	2	499
fib-1	Q22053	rRNA 2'-O-methyltransferase fibrillarin	5	4	3	7	6	6	4	2	0	712
rab-1	Q9UAQ6	Rab family protein 1	4	5	0	3	0	4	4	2	0	758
W08E12.7	Q9N5B3	Putative uncharacterized protein	0	0	0	0	0	0	4	2	0	1063
rps-26	O45499	40S ribosomal protein S26	4	3	3	4	5	3	4	2	2	1335

rpl-19	O02639	60S ribosomal protein L19	9	6	4	4	12	5	4	3	0	3055
tag-210	P91917	Putative GTP-binding protein tag-210	3	0	3	0	0	4	4	3	0	476
rpl-14	Q9XVE9	Protein C04F12.4, confirmed by transcript evidence	0	5	5	4	5	4	4	3	2	3227
cco-2	P55954	Cytochrome c oxidase subunit 5A, mitochondrial (Cytochrome c oxidase polypeptide	0	0	2	0	0	2	4	3	2	916
rps-7	Q23312	40S ribosomal protein S7	2	5	11	8	11	6	4	4	0	2334
tufm-1	Q19072	Elongation factor Tu	4	4	0	6	5	2	4	4	0	330
hsp-12.2	P34328	Heat shock protein Hsp-12.2	0	0	0	0	0	0	4	4	0	412
vig-1	O16646	Vig (Drosophila vasa intronic gene) ortholog protein 1	5	7	7	6	5	4	4	4	2	1843
ldh-1	Q27888	L-lactate dehydrogenase	4	6	0	6	2	6	4	4	2	279
let-767	C1P622	Lethal protein 767, isoform b, confirmed by transcript evidence	2	2	2	2	0	7	4	4	2	184
gst-6	P91252	Probable glutathione S-transferase 6	2	4	0	0	2	0	4	4	2	122
K08E3.5	Q69Z13	Protein K08E3.5f, confirmed by transcript evidence	0	0	0	0	0	0	4	4	2	345
T22F3.3	Q86NC1	Phosphorylase	6	3	3	9	7	2	4	5	0	970
mca-3	Q95XP6	Putative uncharacterized protein	2	0	0	8	0	0	4	5	0	153
rps-2	P51403	40S ribosomal protein S2	2	9	8	4	7	3	4	5	0	2158
T25B9.9	Q17761	6-phosphogluconate dehydrogenase	0	4	0	2	0	0	4	5	0	557
rpn-2	Q18115	26S proteasome non-ATPase regulatory subunit 1	4	3	3	10	4	5	4	5	2	184
ucr-2.1	Q9BI61	Protein VW06B3R.1b, confirmed by transcript evidence	2	4	4	2	0	0	4	5	2	968
aldo-1	P54216	Fructose-bisphosphate aldolase 1	5	3	4	4	2	2	4	5	3	2067
hel-1	Q18212	Spliceosome RNA helicase DDX39B homolog	0	0	2	0	2	3	4	5	3	337
rpl-24.1	O01868	60S ribosomal protein L24	0	7	0	4	10	8	4	6	0	3526
tcp-1	P41988	T-complex protein 1 subunit alpha	0	4	4	2	2	0	4	6	0	472
rps-8	P48156	40S ribosomal protein S8	6	11	7	11	11	9	4	6	3	2237
rps-19	O18650	40S ribosomal protein S19	4	7	7	0	6	5	4	6	3	2483
F47B10.1	P53588	Succinyl-CoA synthetase beta-A chain	5	4	5	2	5	6	4	7	2	695
irs-1	Q21926	Isoleucyl-tRNA synthetase	4	2	3	9	0	3	4	8	0	122
cat-2	O61235	Catalase-2	0	0	0	0	0	2	4	8	0	3.23
rpl-21	P34334	60S ribosomal protein L21	2	6	3	5	8	5	4	8	3	2356
Y37E3.17	Q6AW03	Putative uncharacterized protein	0	2	0	7	0	0	4	10	3	310
cdc-48	P54812	Transitional endoplasmic reticulum ATPase homolog 2	5	2	5	9	4	6	4	11	2	436
unc-15	P10567	Paramyosin (Uncoordinated protein 15)	3	6	5	12	5	5	4	17	6	2391
F22F7.1	Q9GZE9	Putative uncharacterized protein	0	0	0	0	0	0	5	0	0	201
dim-1	Q18066	Disorganized muscle protein 1 (2D-page protein spot 8)	0	0	0	0	0	4	5	0	2	594
rps-14	P48150	40S ribosomal protein S14	2	4	5	4	6	3	5	3	0	1597
cth-1	O45391	Protein F22B8.6, confirmed by transcript evidence	2	0	0	2	0	0	5	3	0	71.2
rpl-6	P47991	60S ribosomal protein L6	7	11	9	6	8	7	5	3	2	2295
acdh-7	Q22781	Acyl coa dehydrogenase protein 7, confirmed by transcript evidence	5	2	3	3	0	4	5	3	2	600
R05G6.7	Q21752	Probable voltage-dependent anion-selective channel	0	6	2	0	5	8	5	3	2	2361
rpl-8	Q9XVF7	60S ribosomal protein L8	5	10	6	9	5	8	5	3	3	1650
unc-60	Q07750	Actin-depolymerizing factor 1, isoforms a/b (Uncoordinated protein 60)	2	3	0	0	0	0	5	3	3	1788
rps-9	Q20228	40S ribosomal protein S9	0	9	5	4	6	4	5	4	0	2186
glrx-10	Q9N456	Glutaredoxin protein 10	3	4	5	2	5	2	5	4	4	684
F41C3.5	P52717	Uncharacterized serine carboxypeptidase F41C3.5	4	4	3	0	2	3	5	5	0	630
gst-38	O45451	Protein F35E8.8, confirmed by transcript evidence	2	3	0	3	0	0	5	5	2	8.02
asb-2	Q19126	Atp synthase b homolog protein 2	4	6	2	5	0	6	5	5	4	557
phb-1	Q9BKU4	Mitochondrial prohibitin complex protein 1 (Prohibitin-1)	3	2	6	2	3	6	5	5	4	1108
rpl-7	O01802	60S ribosomal protein L7	8	10	6	8	8	13	5	5	5	2403
atp-5	Q17763	Protein C06H2.1, confirmed by transcript evidence	10	8	7	3	2	5	5	5	5	1427
T02G5.7	Q22101	Putative uncharacterized protein	0	0	0	3	0	0	5	6	0	480
glb-1	P30627	Globin-like protein	0	2	2	0	2	0	5	6	0	653
sams-1	O17680	PS-adenosylmethionine synthase 1	2	0	0	0	0	2	5	6	0	672
act-5	O45815	Protein T25C8.2, confirmed by transcript evidence	6	4	3	4	4	2	5	6	5	1298
imb-3	Q9N5V3	Importin beta family protein 3, confirmed by transcript evidence	5	5	7	9	4	9	5	7	0	121
LLC1.3	O17953	Protein LLC1.3a, confirmed by transcript evidence	2	3	0	5	0	0	5	7	0	984
cct-8	Q9N358	T-complex protein 1 subunit theta (TCP-1-theta) (CCT-theta)	4	2	3	2	0	2	5	7	0	473
F53A2.7	O45552	Protein F53A2.7, confirmed by transcript evidence	4	0	3	3	0	3	5	7	2	1165
prdx-2	A8DYR6	Peroxiredoxin protein 2	5	7	5	4	6	2	5	9	2	2916
rps-20	Q8WQA8	Protein Y105E8A.16, confirmed by transcript evidence	3	4	3	0	6	3	5	10	3	3421
vha-15	Q22494	Vacuolar proton pump subunit H 2	5	5	2	7	6	4	5	10	5	416
hsp-60	P50140	Chaperonin homolog Hsp-60, mitochondrial (Heat shock protein 60)	0	0	0	0	0	0	5	11	0	2423
alh-9	P46562	Putative aldehyde dehydrogenase family 7 member A1 homolog	3	3	0	3	2	0	5	11	3	601
cct-6	P46550	T-complex protein 1 subunit zeta	4	6	0	0	0	0	5	12	2	472
T25F10.6	Q23050	Putative uncharacterized protein T25F10.6	6	3	0	9	0	2	5	15	4	1392
T22B11.5	O61199	Alpha-ketoglutarate dehydrogenase	10	7	14	17	8	12	5	16	5	530
rpl-9	Q95Y90	60S ribosomal protein L9	2	10	5	7	9	4	6	2	0	2896
asg-2	Q18803	Probable ATP synthase subunit g 2	7	5	3	0	3	3	6	2	3	481
phb-2	P50093	Mitochondrial prohibitin complex protein 2 (Prohibitin-2)	4	5	4	5	4	5	6	3	5	604

R07H5.8	Q93934	Protein R07H5.8, confirmed by transcript evidence	2	2	0	3	0	2	6	4	2	1017
acp-6	Q9GUF2	Acid phosphatase family protein 6, confirmed by transcript evidence	8	2	3	6	2	5	6	4	3	220
fum-1	O17214	Probable fumarate hydratase	2	2	0	2	0	0	6	5	0	934
D2063.3	Q6EZG4	Putative uncharacterized protein	2	0	0	2	0	0	6	5	0	93.7
rpl-30	Q9XWS4	Protein Y106G6H.3, confirmed by transcript evidence	4	3	5	0	5	2	6	5	0	1099
sodh-1	Q17334	Alcohol dehydrogenase 1 (Sorbitol dehydrogenase family protein 1)	3	6	4	4	3	3	6	6	2	1763
rps-15	Q9XVP0	40S ribosomal protein S15	3	7	7	5	8	4	6	6	3	2721
rpl-33	P49180	60S ribosomal protein L35a	2	6	6	0	3	2	6	6	5	2326
C16A3.10	Q18040	Probable ornithine aminotransferase	7	4	3	6	5	3	6	7	3	730
rps-12	P49196	40S ribosomal protein S12	0	3	5	0	2	5	6	8	6	900
rpl-4	O02056	60S ribosomal protein L4	17	18	10	22	19	13	6	9	6	2708
gst-41	Q966G8	Glutathione s-transferase protein 41	5	13	4	3	6	4	6	10	4	29.9
rps-1	P48154	40S ribosomal protein S3a	9	10	12	12	13	7	6	11	2	2661
F57B10.3	O44742	Cofactor-independent phosphoglycerate mutase	0	4	3	2	2	2	6	11	3	705
Y43F4B.5	O45934	Protein Y43F4B.5a, confirmed by transcript evidence	0	0	0	0	0	0	6	11	4	330
cct-2	P47207	T-complex protein 1 subunit beta	2	0	0	2	0	2	6	12	0	444
Y71H10A.1	Q9TZL8	6-phosphofructokinase	3	4	4	11	4	6	6	12	2	280
gdh-1	Q23621	Glutamate dehydrogenase	5	6	5	6	3	6	6	16	7	
F17C11.9	P54412	Probable elongation factor 1-gamma (EF-1-gamma) (eEF-1B gamma)	3	0	0	0	0	0	7	3	0	937
rps-11	Q20206	Protein F40F11.1, confirmed by transcript evidence	2	5	8	5	8	6	7	4	3	1776
gpd-1	P04970	Glyceraldehyde-3-phosphate dehydrogenase 1	6	6	2	6	3	6	7	7	0	756
K08D12.3	Q966I7	Putative uncharacterized protein	0	0	0	0	0	0	7	7	2	328
rpa-0	Q93572	60S acidic ribosomal protein P0	8	5	8	6	3	10	7	8	7	2663
C37E2.1	Q93353	Isocitric dehydrogenase subunit beta	5	5	4	3	0	5	7	10	5	338
enol-1	Q27527	2-phospho-D-glycerate hydro-lyase	4	12	3	6	7	2	7	11	0	4422
rpl-7A	Q966C6	60S ribosomal protein L7a	8	12	9	12	7	10	7	11	5	2015
vha-12	Q19626	Vacuolar proton pump subunit B	6	6	4	7	5	4	7	12	4	1979
pccb-1	Q20676	Putative uncharacterized protein	5	6	5	9	5	0	7	17	4	846
pam-1	Q20627	Puromycin-sensitive aminopeptidase protein 1, isoform a	6	3	3	7	7	5	7	18	3	418
gei-7	Q10663	Bifunctional glyoxylate cycle protein (Gex-3-interacting protein 7)	19	5	10	23	16	13	7	18	6	777
F58F12.1	Q09544	ATP synthase subunit delta, mitochondrial (F-ATPase delta subunit)	2	3	0	3	0	2	8	3	3	1959
rpl-18	O45946	60S ribosomal protein L18	7	12	6	9	10	9	8	5	0	3797
mdh-1	Q9UAV5	Malate dehydrogenase	5	3	3	4	3	0	8	5	0	2090
rpl-12	P61866	60S ribosomal protein L12	3	5	4	6	4	3	8	6	2	3385
F43G9.1	Q93714	Probable isocitrate dehydrogenase	7	5	6	3	3	6	8	7	0	802
cct-3	Q9N4J8	Putative uncharacterized protein	2	2	0	3	0	3	8	8	0	385
rps-4	Q9N3X2	40S ribosomal protein S4	11	19	16	22	16	19	8	9	2	2657
Y69A2AR	Q95XJ0	ATP synthase gamma chain	8	9	8	11	9	7	8	9	4	1322
C44B7.10	Q18599	Putative uncharacterized protein	9	9	8	9	9	7	8	9	5	2154
rps-16	Q22054	40S ribosomal protein S16	9	9	10	8	12	11	8	9	8	2456
acdh-12	Q19057	Acyl coa dehydrogenase protein 12, isoform a	0	2	0	0	0	0	8	10	4	491
T08B2.7	Q9BIC3	Putative uncharacterized protein	3	2	15	14	11	11	8	12	5	479
gst-39	Q9NAB0	Protein Y53F4B.33, confirmed by transcript evidence	16	14	7	9	6	4	8	12	7	109
F49E2.2	A6ZJ46	Protein F49E2.2c, partially confirmed by transcript evidence	5	8	0	7	0	0	8	13	4	58.9
hsp-3	P27420	Heat shock 70 kDa protein C	6	7	0	6	0	3	8	15	4	1510
pyk-1	B7WNA0	Protein F25H5.3e, partially confirmed by transcript evidence	4	4	0	4	0	0	8	16	0	443
W10C8.5	O45011	Putative uncharacterized protein	3	4	3	3	0	0	9	9	3	306
ucr-1	P98080	Ubiquinol-cytochrome-c reductase complex core protein 1	7	10	6	8	7	9	9	11	7	1192
fln-1	D0IMZ5	FLN-1 protein, isoform a, partially confirmed by transcript evidence	7	3	9	17	4	12	9	16	4	343
tct-1	Q93573	Translationally-controlled tumor protein homolog (TCTP)	2	6	5	2	0	2	10	0	2	3680
gst-27	Q9NAB3	Protein Y53F4B.30, confirmed by transcript evidence	15	7	5	8	7	3	10	10	4	355
mlc-1	P19625	Myosin regulatory light chain 1	5	6	6	6	7	5	10	10	6	2151
aco-1	Q23500	Probable cytoplasmic aconitate hydratase (Aconitase)	6	6	6	9	3	4	10	19	5	882
gst-26	Q9NAB4	Protein Y53F4B.29, confirmed by transcript evidence	9	8	6	6	4	4	11	7	5	318
inf-1	P27639	Eukaryotic initiation factor 4A (eIF-4A)	9	7	3	9	6	5	11	10	5	735
F01G4.6	P40614	Phosphate carrier protein, mitochondrial (PTP)	9	7	10	11	8	8	11	12	9	613
rpl-5	P49405	60S ribosomal protein L5	10	15	8	16	12	11	11	13	8	3694
rack-1	Q21215	Guanine nucleotide-binding protein subunit beta-2-like 1	8	10	7	7	7	9	11	14	3	2329
gst-13	Q22814	Protein T26C5.1, confirmed by transcript evidence	16	18	10	7	13	8	11	16	11	136
tkt-1	O17759	Protein F01G10.1, confirmed by transcript evidence	10	4	4	6	0	3	11	19	3	1834
T25G3.4	P90795	Probable glycerol-3-phosphate dehydrogenase	7	5	3	7	0	3	11	20	2	159
F46H5.3	Q10454	Probable arginine kinase F46H5.3 (AK) (EC 2.7.3.3)	7	7	6	6	4	5	12	8	4	4094
C08H9.2	Q17832	Protein C08H9.2a, confirmed by transcript evidence	4	5	9	13	6	7	12	11	2	583
rps-0	P46769	40S ribosomal protein SA	10	11	10	11	9	10	12	12	8	2456
gst-4	Q21355	Glutathione S-transferase 4	11	16	6	6	13	5	12	13	6	83
F45D11.15	Q9N2K4	Putative uncharacterized protein	8	12	7	8	5	4	12	15	0	716
mlc-3	P53014	Myosin, essential light chain (Myosin light chain alkali)	13	12	6	6	7	8	12	16	11	7584

vha-13	Q9XW92	Vacuolar proton pump subunit alpha	7	7	0	5	0	2	12	17	6	2335
aco-2	P34455	Probable aconitate hydratase (Aconitase)	12	8	13	18	10	10	12	19	8	1933
gpd-2	P17329	Glyceraldehyde-3-phosphate dehydrogenase 2 (GAPDH-2)	9	9	6	10	5	4	13	10	6	2973
rps-3	P48152	40S ribosomal protein S3	10	13	11	14	12	12	13	10	8	2815
hsp-6	P11141	Heat shock 70 kDa protein F, mitochondrial	4	5	0	5	0	0	13	14	4	1261
chc-1	P34574	Probable clathrin heavy chain 1	20	11	8	29	16	9	13	15	5	247
5C820	Q9GQ62	5C820	4	8	5	0	5	2	14	14	8	342
gst-28	Q9NAB2	Protein Y53F4B.31, confirmed by transcript evidence	17	12	6	18	12	8	14	16	6	174
sca-1	Q9XU13	Protein K11D9.2b, confirmed by transcript evidence	18	10	8	21	12	9	14	21	4	634
**dep-1**	**Q20120**	**Protein F44G4.8a, partially confirmed by transcript evidence**	**68**	**55**	**51**	**66**	**42**	**48**	**14**	**23**	**3**	**1.16**
eat-6	P90735	Protein B0365.3, confirmed by transcript evidence	18	10	11	23	12	13	14	24	5	426
idh-1	Q21032	Isocitrate dehydrogenase	8	9	0	9	6	0	15	10	0	1244
gst-36	Q09607	Probable glutathione S-transferase gst-36	14	16	9	9	8	10	15	11	9	375
mdh-1	O02640	Probable malate dehydrogenase	11	9	7	10	4	8	15	15	7	2090
pcca-1	Q19842	Propionyl-CoA carboxylase alpha chain	16	8	8	26	12	10	15	18	3	902
ahcy-1	P27604	Adenosylhomocysteinase (AdoHcyase)	10	12	8	13	9	8	16	11	6	4696
sdha-1	Q09508	Succinate dehydrogenase	8	10	2	8	0	5	16	16	9	635
tba-2	P34690	Tubulin alpha-2 chain	14	18	12	19	17	15	17	17	5	874
gst-20	O01987	Protein Y48E1B.10 gst-20	16	23	13	12	19	11	17	18	10	266
pyc-1	O17732	Pyruvate carboxylase 1	11	11	8	26	15	10	17	19	2	327
ant-1	O45865	Protein T27E9.1a, confirmed by transcript evidence	12	18	15	16	18	19	18	23	15	819
daf-21	Q18688	Heat shock protein 90 (Abnormal dauer formation protein 21)	20	15	18	30	17	25	19	29	10	1522
hsp-1	P09446	Heat shock 70 kDa protein A	13	16	9	15	2	13	20	28	13	2550
gst-5	Q09596	Probable glutathione S-transferase 5	19	23	14	15	18	15	21	19	14	199
gst-7	P91253	Probable glutathione S-transferase 7	23	23	14	13	18	16	21	24	18	778
W05G11.6	O44906	Putative uncharacterized protein W05G11.6	22	17	13	20	2	12	22	32	6	1439
tbb-2	P52275	Tubulin beta-2 chain (Beta-2-tubulin)	19	24	16	31	21	25	23	21	10	1372
gst-1	P10299	Glutathione S-transferase P	22	24	21	21	17	21	24	23	18	690
eef-2	P29691	Elongation factor 2 (EF-2)	20	6	10	26	19	16	24	34	13	7.81
vit-5	P06125	Vitellogenin-5	69	50	42	167	129	85	24	47	9	750
gst-10	Q9N4X8	Glutathione S-transferase P 10	25	25	17	16	24	20	25	28	20	215
R11A5.4	O02286	Protein R11A5.4a, confirmed by transcript evidence	19	22	14	22	3	13	25	34	19	1849
atp-2	P46561	ATP synthase subunit beta, mitochondrial	26	21	18	29	17	21	26	24	18	6267
eft-3	P53013	Elongation factor 1-alpha (EF-1-alpha)	19	22	21	20	21	16	28	27	15	3510
act-1	P10983	Actin-1/3	25	25	18	29	21	18	29	23	17	2359
H28O16.1	Q9XXK1	ATP synthase subunit alpha, mitochondrial	26	26	24	32	25	18	31	38	25	6282
myo-1	P02567	Myosin-1 (Lethal protein 75)	30	14	7	39	17	11	32	67	27	n.a.
myo-3	P12844	Myosin-3 (Myosin heavy chain A)	37	10	2	49	8	7	35	44	14	489
vit-2	P05690	Vitellogenin-2	78	54	55	184	123	86	37	55	14	1514
myo-2	P12845	Myosin-2 (Myosin heavy chain C)	33	21	2	50	24	12	39	79	31	570
vit-6	P18948	Vitellogenin-6	102	68	87	227	177	152	56	82	33	1565
myo-4	P02566	Myosin-4 (Myosin heavy chain B) (Uncoordinated protein 54)	130	71	69	162	62	85	126	175	113	n.a.

Supplementary table 1

Numbers of peptides per protein identified by LC-MS/MS analyses in the DEP-1 intra_wt, DEP-1 intra_DA, and GST pull-downs. LC-MS/MS analyses identified 585 proteins. Every pull-down was done in triplicates. The numbers represent the peptides that were detected. Min. Protein Probability = 95%; Min. Number of Peptides = 2. Values from „Protein abundance“ correspond to the „*C.elegans* PaxDB integrated dataset“ (www.pax-db.org).
