# Supplementary material for: β-Integrin de-phosphorylation by the Density-Enhanced Phosphatase DEP-1 attenuates EGFR signaling in C. elegans
Source: PLoS Genet. 2017 Jan 30;13(1):e1006592. doi: 10.1371/journal.pgen.1006592 (PMC5305270; doi:10.1371/journal.pgen.1006592)
Supplement: S2 Table — Potential off-target sites of pat-3 sgRNA #2 and pat-3 sgRNA #4, which were rated by the CRISPR Design Tool at www.crispr.mit.edu [29] with an “off-target hit score” ≥0.2, were PCR amplified and sequenced with the indicated forward- and reverse primers. None of the analyzed off-target sites were altered by the CRISPR/CAS9-mediated genome editing. For tln-1 sgRNA #4 no off-target sites with a score ≥0.2 were predicted. (PDF) [file pgen.1006592.s002.pdf]

S2 Table

pat-3 **sgRNA #2 ATGGGATACGGTAAGTGACA CGG**
on-target locus: chrIII:+3909295

Possible Off-target Sequence	Off-target Hit Scores	Mismatches	Chromosome	Gene name	Forward Primer		Reverse Primer		Sequencing
ATGTGATGCGGTGAGTGACATGG	1.1	3MMs [4:8:13]	II	gcy-19	OMW520	GGAGATATAGGCCACCTGTCAAAG	OMW521	GTGGATCAATATGGAGCTCTCGTG	OK
ATGTGAGAGAGTAAGTGACATAG	0.5	4MMs [4:7:9:10]	II	abts-3	OMW522	CTACCAATTCGCAGTCCATCAGTTC	OMW523	CCAGATGTCTTGATCCTAGCGAAC	OK
ATGTGAATCGGGAAGTGACACGG	0.5	4MMs [4:7:8:12]	III	C35D10.8	OMW524	CAACGAACGCAATCTCCTAGTCAC	OMW525	CGGGTGATTTTTCTGACTTGTCCG	OK
ATGCCACACGGTAAGTAACATAG	0.4	4MMs [4:5:7:17]	IV	n.n.	OMW526	GGATGTTCATAAACTCCACAGTATTC	OMW527	GTCAAATGCCCTTCAAGTAGAGGG	OK
ATGGGTTAAGGTAAGTGAGAGAG	0.4	3MMs [6:9:19]	IV	ZK792.1	OMW528	CGAGACCTAAGAGATCACCATTCAC	OMW529	GTAGGTAGTACCTACTAGGCACAG	OK
ATTAGATACGGAAAGTGAAAAGG	0.2	4MMs [3:4:12:19]	I	pgp-2	OMW530	CATTCTAGGATTGCCAGATGTAGG	OMW531	CACTGACGATCGCAATGACGTCAC	OK

pat-3 **sgRNA #4 AAGGATAAAAACTATTTAGT TGG**
on-target locus: chrIII:-3909422

Possible Off-target Sequence	Off-target Hit Scores	Mismatches	Chromosome	Gene name	Forward Primer		Reverse Primer		Sequencing
AAGTATAAAAACTATTAAGTAGG	4.3	2MMs [4:17]	X	C37E2.3	OMW532	GCCCAGAATGGTAGTTCATCATTCC	OMW533	CATCCATGATGTCGAGTTGCTTGG	OK
AAAATTAAAAACAATTTAGTTGG	0.6	4MMs [3:4:5:13]	I	n.n.	OMW534	GGTAACAGTTTAAGCCAAACAGGC	OMW535	GAATGAAACGTTGACTGTTGCCGC	OK
AATGTTTAAAACTATTTATTAAG	0.3	4MMs [3:5:7:19]	IV	n.n.	OMW536	CTTTGGTACCCGGATGTCAGGAAATG	OMW537	GACATTGGAGAATCAGACGGACGAG	OK
GATAATAAAAACTATTTTGTAGG	0.3	4MMs [1:3:4:18]	II	C08G5.1	OMW538	CGGTAATTTCCAGACGTAGTTGATAG	OMW539	GTCTCAAATGGCACAAGAAACAGCG	OK
TAGGACAAAAAGTATTTAGGGGG	0.2	4MMs [1:6:12:20]	V	F53F8.10	OMW540	GTTTGCCGATCACCAATCACTTTGC	OMW541	CAGGCGTTTTGAGACCATCTATCG	OK
TAGAATAAAAACTATTGAATTAG	0.2	4MMs [1:4:17:19]	V	n.n.	OMW542	CAGACGTGTCAGACAAGGATGCAC	OMW543	GATAAGACACGCAAAGGCTGGGC	OK
AAGGCAAAAAAGTATTTAGGGGG	0.2	4MMs [5:6:12:20]	V	n.n.	OMW544	CTTGTATGACTTCCACATCTTGCG	OMW545	CAGCCAGTTGGACAACACATTGAAG	OK
AAATATAAAAACCATTTAATAAG	0.2	4MMs [3:4:13:19]	II	n.n.	OMW546	GTCAAGATTTGTGATTGGTTCTGCG	OMW547	CGGCAACTTGGTTTACGAACCCTG	OK
AGAGAGAAAAACTATTTTGTTGG	0.2	4MMs [2:3:6:18]	III	n.n.	OMW548	CTCGCATCCTTTGAACTTCAAACGC	OMW549	CTAATTTGTCCAGGTACTCCCAGC	OK

Offtarget analysis: www.crispr.mit.edu
