# Supplementary material for: β-Integrin de-phosphorylation by the Density-Enhanced Phosphatase DEP-1 attenuates EGFR signaling in C. elegans
Source: PLoS Genet. 2017 Jan 30;13(1):e1006592. doi: 10.1371/journal.pgen.1006592 (PMC5305270; doi:10.1371/journal.pgen.1006592)
Supplement: S4 Table — (PDF) [file pgen.1006592.s004.pdf]

S4 Table

Primer name	Sequence (5’ - 3’)
OCU3	CAGAACACGGTGAATGCAACTGTG
OCU4	CTCTTGACAGATCGTAAACAAGCGC
OMW288	GAACGTCGTGGTAGCTTGCTTGAAG
OMW111	CTAACGAATCAGCAATCTGTGAGG
OMW102	CATGTCTTCTCAGATAGTTATGGC
OMW233	GCCATAACTATCTGAGAAGACATG
OMW234	CTAATCGTTTGGCACGTATTGAGC
OCU18	GACACCGAACAAAAAACATATAAATTTATC
OCU19	GTTTTTTGTTCGGTGTCACTTACCGTATC
OSN73	GCATCACCTTCACCCTCTCC
OMW112	CCTGTTGAGGAATTACCCAGTAAG
OMW277	GGTTGGCATCTGTTGGAATCTCTAG
OMW266	GGTGTCTGAAGAGAAAATTGATAATAAG
OMW267	CAATTTTCTCTTCAGACACCATGAGTAAAGGAGAAGAACTTTTCAC
OMW268	GACCGTCAGACTGAGGACTCCCATTTTGTATAGTTCATCCATGCCATG
OMW269	ATGGGAGTCCTCAGTCTGACGGTC
OCU44	CTCCTTCTCCTTCAAATTCCACG
OMW265	GGCATATCCCAGAAACTAGTAAGC

S4 Table
Sequences of primers used.
