# Supplementary figures and images for: β-Integrin de-phosphorylation by the Density-Enhanced Phosphatase DEP-1 attenuates EGFR signaling in C. elegans
Source: PLoS Genet. 2017 Jan 30;13(1):e1006592. doi: 10.1371/journal.pgen.1006592 (PMC5305270; doi:10.1371/journal.pgen.1006592)

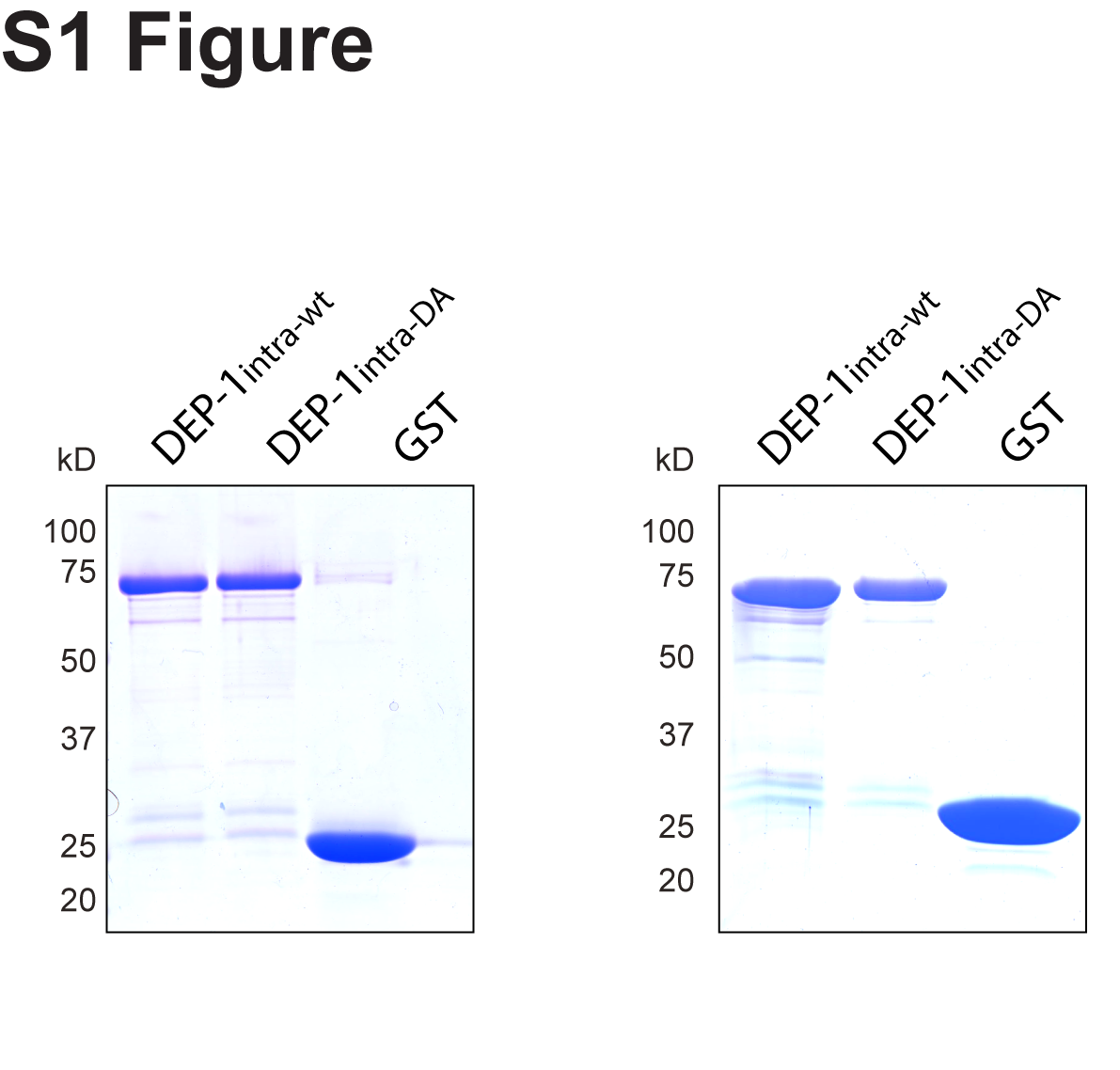

Supplement: S1 Fig — (TIF) [file pgen.1006592.s005.tif]
