# Supplementary material for: β-Integrin de-phosphorylation by the Density-Enhanced Phosphatase DEP-1 attenuates EGFR signaling in C. elegans
Source: PLoS Genet. 2017 Jan 30;13(1):e1006592. doi: 10.1371/journal.pgen.1006592 (PMC5305270; doi:10.1371/journal.pgen.1006592)
Supplement: S1 Methods — (DOCX) [file pgen.1006592.s006.docx]

**Supplementary materials and methods**

**CRISPR/Cas9-mediated genome editing**

Two *pat-3* target sequences near the NPXY_792_ motif (*pat-3 sgRNA #2:* ATGGGATACGGTAAGTGACA and *pat-3 sgRNA #4:* aaggataaaaactatTTAGT), and one *tln-1* target sequence near the start codon (*tln-1 sg#4* CGTCAGACTGAGGACTCCCA) were identified using the CRISPR Design Tool on <http://crispr.mit.edu> and cloned into pDD162 (Addgene).

For generating the *pat-3(Y792F)* mutant *zh105*, a 2-kb fragment of the C-terminal *pat-3* locus was amplified using OCU3 and OCU4 and subcloned into pGEM®-T Easy (Promega). The tyrosine Y_792_ was mutated to phenylalanine F_792_, and the PAM motif corresponding to *pat-3* *sgRNA #2* was mutated by site directed mutagenesis. Additionally, a primer binding site polymorphism (PBSP) consisting of the silent mutations in K_793_, Q_794_, A_795_, T_796_, T_797_, T_798_, and T_799_ was inserted by site directed mutagenesis. Using the co-conversion strategy described by [50], 90ng/µl of the *pat-3(Y_792_F)* donor template plasmid were microinjected together with 100 ng/µl of *pat-3 sgRNA #2,* 50ng/µl *dpy-10 sgRNA,* 50ng/µl CAS9 plasmid pDD162 and 500nM *dyp-10* donor template oligonucleotide into wild-type N2 animals*.* Three days after the injection, single F1 recombinants showing a Dpy or Rol phenotype were transferred to individual NGM plates and allowed to produce F2 progeny for 2–3 days. Genomic DNA was then prepared by lysis of young adult F2 hermaphrodites, and *pat-3(Y792F)* mutants were identified by PCR amplification using the primer binding site polymorphism specific primer OMW288 and OMW111.

For the CRISPR/Cas9 generated endogenous *pat-3::GFP* reporters *pat-3(zh115)* (wild-type) and *pat-3(zh116)* (Y792F mutant), a 2.8-kb fragment was PCR-amplified from the plasmid containing *pat-3::GFP* (see above) with OMW111 and OMW102. The amplicon was then fused with the PCR product amplified from genomic N2 DNA with OMW233 and OMW234 by PCR-fusion and subcloned into into pGEM®-T Easy (Promega). The PAM site corresponding to *pat-3* sgRNA #2 was mutagenized by site directed mutagenesis with the primers OCU18 and OCU19. In the donor plasmid to generate the mutant *zh116* reporter, Y_792_ was further mutated to phenylalanine F_792_. One-hundred ng/µl of the wild-type or mutant *pat-3::GFP* donor template plasmid were injected together with 100 ng/µl of *pat-3 sg#4,* 50ng/µl *dpy-10 sgRNA,* 50ng/µl CAS9 plasmid pDD162 and 500nM *dyp-10* donor template oligonucleotide into wild-type N2 animals. Genomic DNA was prepared by lysis of young adult F2 hermaphrodites as described above and *pat-3::GFP* mutants were identified by PCR amplification using the primers OSN73 and OMW112.

For the CRISPR/Cas9 generated endogenous *GFP::tln-1* reporter *tln-1(zh117)*, a 800-bp fragment was amplified from genomic N2 DNA with OMW277 and OMW266. The amplicon was fused by PCR-fusion to a 900-bp fragment that was amplified from pPD95.75 with OMW267 and OMW268, and to a 660-bp fragment that was amplified from genomic N2 DNA with OMW269 and OCU44. The resulting amplicon was subcloned into pCR-Blunt II-TOPO (Thermofisher). 100 ng/µl of the *GFP::tln-1* donor template plasmid were injected together with 100 ng/µl of *tln-1 sgRNA #4,* 50ng/µl *dpy-10 sgRNA,* 50ng/µl CAS9 plasmid pDD162 and 500nM *dyp-10* donor template oligonucleotide into wild-type N2 animals. Genomic DNA was prepared by lysis of young adult F2 hermaphrodites, and *GFP::tln-1* mutants were identified by PCR amplification using the primers OSN73 and OMW265.
